# Proteome and Phosphoproteome of Tomato Fruit Identify REDUCED CHLOROPLAST COVERAGE 1a as A Ripening Regulator

**DOI:** 10.1093/gpbjnl/qzaf050

**Published:** 2025-06-09

**Authors:** Jinjuan Tan, Zhongjing Zhou, Hanqian Feng, Jiateng Zhang, Ruikai Zhang, Zhongkai Chen, Yujie Niu, Fangyu Liu, Zhiping Deng

**Affiliations:** Key Laboratory of Traceability for Agricultural Genetically Modified Organisms, Ministry of Agriculture and Rural Affairs, Institute of Virology and Biotechnology, Zhejiang Academy of Agricultural Sciences, Hangzhou 310021, China; Key Laboratory of Traceability for Agricultural Genetically Modified Organisms, Ministry of Agriculture and Rural Affairs, Institute of Virology and Biotechnology, Zhejiang Academy of Agricultural Sciences, Hangzhou 310021, China; Key Laboratory of Traceability for Agricultural Genetically Modified Organisms, Ministry of Agriculture and Rural Affairs, Institute of Virology and Biotechnology, Zhejiang Academy of Agricultural Sciences, Hangzhou 310021, China; Key Laboratory of Traceability for Agricultural Genetically Modified Organisms, Ministry of Agriculture and Rural Affairs, Institute of Virology and Biotechnology, Zhejiang Academy of Agricultural Sciences, Hangzhou 310021, China; College of Life Sciences, Hebei Normal University, Shijiazhuang 050024, China; Key Laboratory of Traceability for Agricultural Genetically Modified Organisms, Ministry of Agriculture and Rural Affairs, Institute of Virology and Biotechnology, Zhejiang Academy of Agricultural Sciences, Hangzhou 310021, China; College of Life Sciences, Hebei Normal University, Shijiazhuang 050024, China; Key Laboratory of Traceability for Agricultural Genetically Modified Organisms, Ministry of Agriculture and Rural Affairs, Institute of Virology and Biotechnology, Zhejiang Academy of Agricultural Sciences, Hangzhou 310021, China; College of Agronomy, Hunan Agricultural University, Changsha 410128, China; Key Laboratory of Traceability for Agricultural Genetically Modified Organisms, Ministry of Agriculture and Rural Affairs, Institute of Virology and Biotechnology, Zhejiang Academy of Agricultural Sciences, Hangzhou 310021, China; Key Laboratory of Traceability for Agricultural Genetically Modified Organisms, Ministry of Agriculture and Rural Affairs, Institute of Virology and Biotechnology, Zhejiang Academy of Agricultural Sciences, Hangzhou 310021, China; Key Laboratory of Traceability for Agricultural Genetically Modified Organisms, Ministry of Agriculture and Rural Affairs, Institute of Virology and Biotechnology, Zhejiang Academy of Agricultural Sciences, Hangzhou 310021, China

**Keywords:** *Solanum lycopersicum*, Carotenoid, Phosphorylation, Plastid, TMT

## Abstract

Fruit ripening in tomato (*Solanum lycopersicum*) has been extensively studied at the transcriptomic level. However, comprehensive profiling of the tomato fruit proteome and phosphoproteome remains limited. In this study, we performed large-scale proteome and phosphoproteome profiling of tomato (Ailsa Craig) fruits across five ripening stages using tandem mass tags (TMT)-based quantitative proteomics. Our analysis quantified over 8800 proteins and 20,000 high-confidence phosphorylation sites. Ripening-associated phosphorylation and dephosphorylation events were identified in diverse ripening regulators, including transcription factors, ethylene biosynthesis and signaling proteins, and epigenetic modifiers. Weighted gene co-expression network analysis (WGCNA) revealed a tetratricopeptide repeat protein, REDUCED CHLOROPLAST COVERAGE 1a (REC1a), as a key regulator of fruit ripening. Parallel reaction monitoring (PRM)-based targeted proteomic analysis validated the expression profiles of REC1a and its three phosphorylation sites. Clustered regularly interspaced short palindromic repeats (CRISPR)-CRISPR-associated protein 9 (Cas9)-mediated knockout of REC1a resulted in reduced lycopene accumulation and slower chlorophyll degradation, highlighting its role in the chloroplast-to-chromoplast transition, which is critical for fruit pigmentation during ripening. Quantitative proteomic analyses of *rec1a* mutants demonstrated reduced levels of Clp proteases and chaperones, proteins known to regulate plastid transitions. Additionally, co-immunoprecipitation and split-luciferase complementation assays revealed that REC1a interacts with the eukaryotic translation initiation factor subunits eIF2α and eIF2Bβ, suggesting its role in regulating protein synthesis during ripening. This study provides the most comprehensive quantitative proteome and phosphoproteome atlas of tomato fruits to date and identifies REC1a as a regulator of fruit ripening, offering new insights into the underlying molecular mechanisms.

## Graphical abstract

**Figure:**
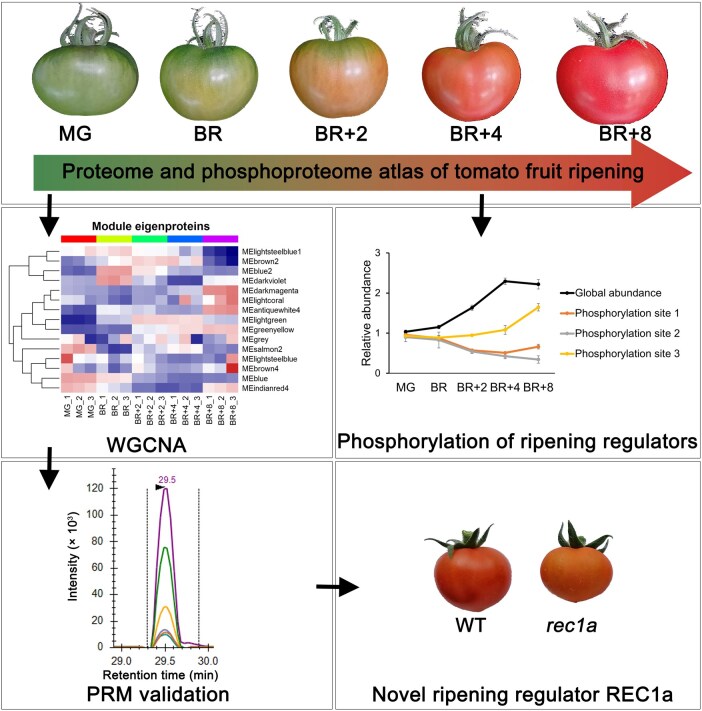

## Introduction

Tomato (*Solanum lycopersicum*), one of the most economically important vegetable crops globally, serves as a model organism for studying fleshy fruit biology. This is due to its relatively small genome size, diploid genetics, short generation time, and ease of genetic transformation [1,2]. Tomato fruit ripening, which begins after seed maturation, is a complex process involving numerous biochemical and physiological changes. These include color transformation, fruit softening, and the accumulation of sugars and volatile compounds [1,2]. These processes are driven by the spatiotemporal regulation of thousands of genes through multiple layers, including epigenetic modifications, transcription, post-transcription, translation, and post-translational modifications (PTMs) [3–5].

Among PTMs, protein phosphorylation is one of the most extensively studied due to its critical role in modulating protein function. Reversible phosphorylation regulates protein stability, localization, activity, and interactions, thereby enhancing the complexity and flexibility of cellular signal transduction. This regulation is vital for plant development and defense against biotic and abiotic stresses [6]. In the context of fruit ripening, several transcription factors (TFs) are regulated by phosphorylation and dephosphorylation. Examples include tomato dehydration-responsive element binding protein 2F (DREB2F) [7], banana MabZIP21 [8], and apple MdbHLH3, MdMYB1, and MdERF17 [9–11]. Additionally, the biosynthesis and signal transduction of ethylene, a critical hormone in tomato ripening, are regulated by phosphorylation. Key examples are 1-aminocyclopropane-1-carboxylic acid synthase (ACS) [12,13], as well as ethylene receptors ETR4 and never ripe (NR) [14]. While large-scale omics data have enabled complex biological network analyses and facilitated functional gene discovery, the coverage of proteins identified through proteomics or PTM-omics studies remains limited in plants. For instance, a comprehensive proteomic analysis of ripening tomato fruits achieved only 19% coverage of annotated protein-coding genes [5]. Moreover, phosphoproteomic profiling of tomato fruits during ripening has not been extensively explored. Therefore, expanding the identification coverage of the proteome and phosphoproteome is crucial for uncovering functional proteins and biological networks involved in fruit ripening.

Tetratricopeptide repeat (TPR) domain proteins are known to regulate diverse biological processes by facilitating protein–protein interactions and protein complex assembly [15,16]. Notably, TPR-containing proteins play roles in chloroplast-associated processes, including messenger RNA (mRNA) processing and stability [17,18], as well as protein assembly and translocation [19–21]. Members of the Reduced chloroplast coverage (REC) and Reduced carotenoid pigmentation 2 (RCP2) protein families, which contain TPR domains, are categorized into four clades in angiosperms, with one or two members per clade. Functional divergence has been observed among these clades [22,23]. In *Arabidopsis*, REC proteins regulate chloroplast compartment size in leaves [22], while RCP2 in monkeyflower (*Mimulus*) is essential for chromoplast development in floral tissues. Downregulation of *RCP2* and its paralogs leads to reduced chlorophyll content and chloroplast coverage in leaves [23]. These findings suggest that REC proteins play conserved roles in plastid function. Recently, the tomato *REC2* gene was identified as a positive regulator of cold tolerance [24].

In this study, we performed large-scale proteomic and phosphoproteomic profiling of tomato fruits across five ripening stages, providing, to our knowledge, the most comprehensive tomato fruit (phospho)proteomic dataset to date. We identified REC1a, a member of the REC1 clade, as a key hub protein in ripening regulation networks at both the protein abundance and phosphorylation levels. Functional characterization of REC1a revealed its critical role in tomato fruit ripening, including plastid development and pigment accumulation. Proteomic analyses of the *rec1a* mutant elucidated the biological processes associated with its phenotype and provided insights into potential molecular mechanisms. Furthermore, we demonstrated that REC1a interacts with eukaryotic translation initiation factor subunits eIF2α and eIF2Bβ, highlighting a possible role in the synthesis of ripening-related proteins.

## Results

### Proteome and phosphoproteome profiling of tomato fruits across ripening stages

**Figure 1:**
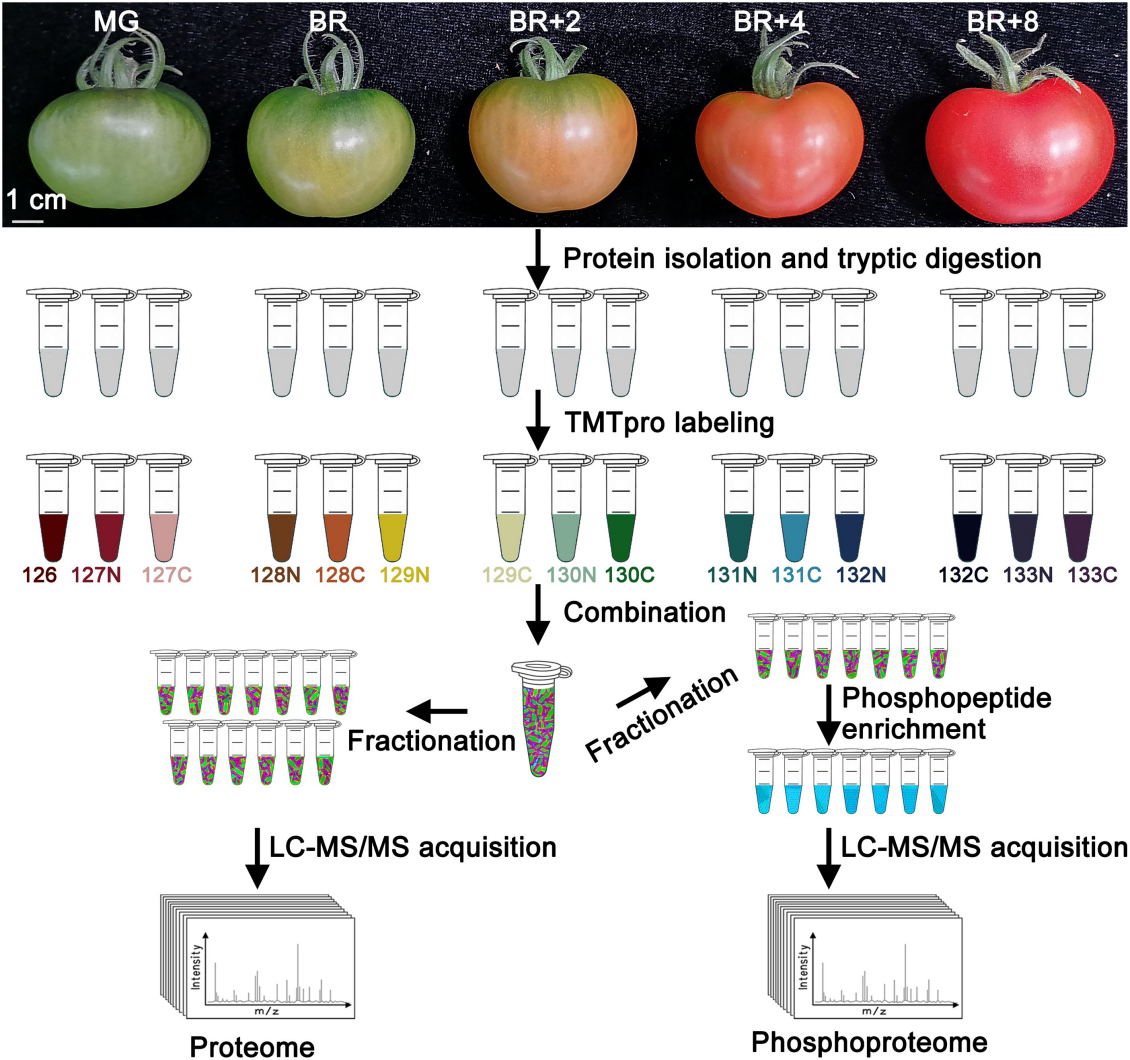
Experimental workflow of stage-resolved tomato proteome and phosphoproteome analyses MG, mature green; BR, breaker; BR+2, breaker + 2 days; BR+4, breaker + 4 days; BR+8, breaker + 8 days; TMTpro, tandem mass tags pro; LC-MS/MS, liquid chromatography with tandem mass spectrometry.

Using a multiplexed isobaric tandem mass tags (TMT)-based quantitative proteomics approach (**Figure 1**), we quantified the proteome and phosphoproteome of ripening tomato (Ailsa Craig) fruit pericarp tissues at five ripening stages: mature green (MG), breaker (BR), breaker + 2 days (BR+2), breaker + 4 days (BR+4), and breaker + 8 days (BR+8). Each stage included three biological replicates (Table S1). The identified proteins in this study refer to protein groups, as certain peptides are shared among multiple proteins or protein isoforms.

**Figure 2:**
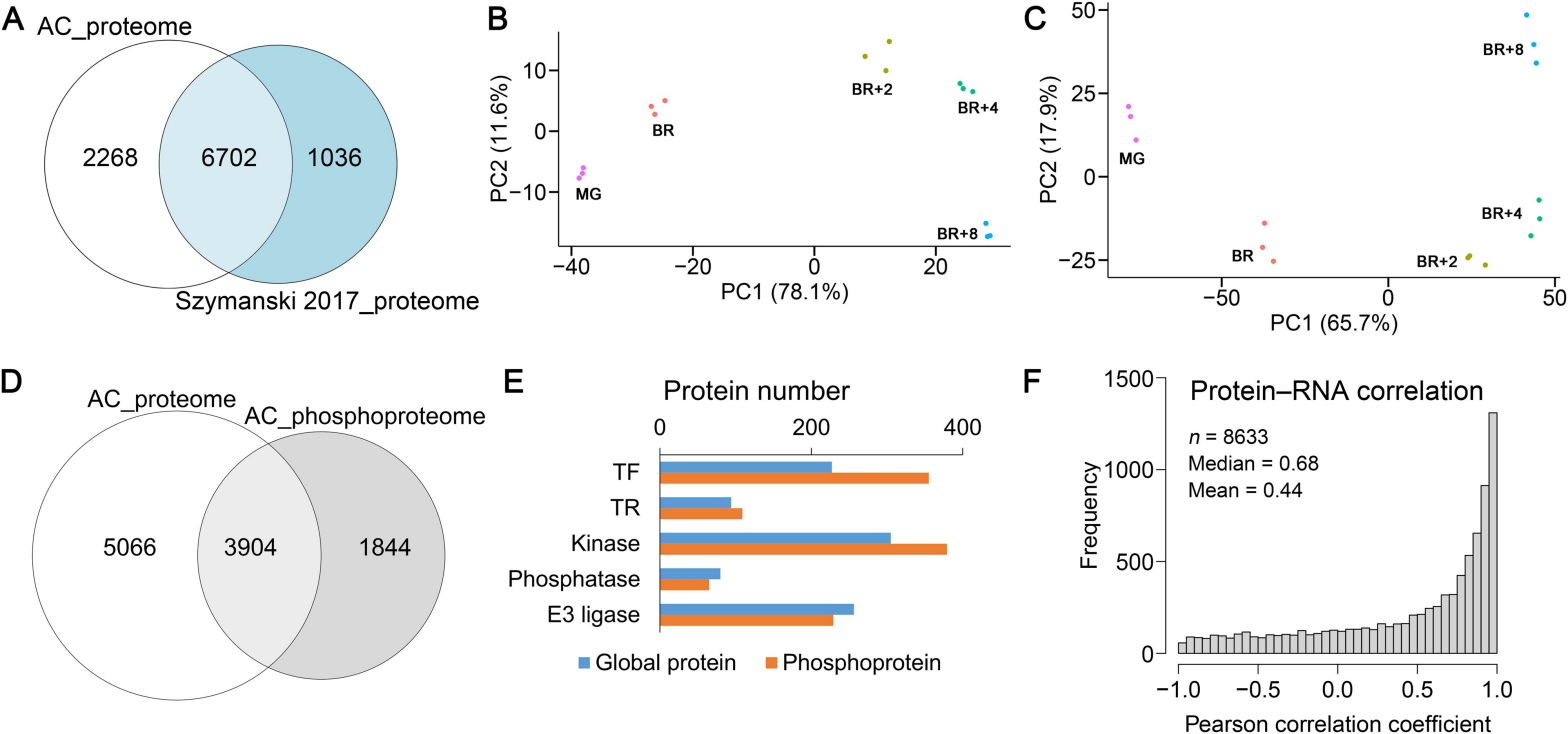
Quantitative global proteome and phosphoproteome analyses of tomato (AC) fruits during ripening **A**. Comparison of protein identification in this study with a prior comprehensive tomato fruit proteome study [5]. **B**. and **C**. PCA of the global proteome (B) and phosphoproteome (C) datasets. **D**. Comparison of protein identification between the proteome and phosphoproteome of AC. **E**. Protein numbers of different categories identified from the fruit proteome and phosphoproteome. **F**. Correlation analysis of protein abundance profiles in this study and transcriptional levels from a previous transcriptome study of tomato fruit [4]. AC, Ailsa Craig; TF, transcription factor; TR, transcription regulator; PC, principal component; PCA, principal component analysis.

A total of 8970 protein groups were identified from global proteome analysis [global false discovery rate (FDR) < 1%], with 8888 quantified across all 15 samples (Table S1). Notably, 25.3% (2268) of the identified proteins were not reported in previous tomato fruit proteome studies [5] (**Figure  2**A). For the phosphoproteome, we identified 31,172 phosphopeptide isoforms, corresponding to 20,829 phosphorylation sites (p-sites) with a ptmRS probability > 75%, representing 5994 protein groups. Among these, 28,908 phosphopeptides were quantified across all samples (Table S1), significantly expanding the scope of p-site identification in tomato fruit proteins.

Most of the identified phosphopeptides were mono-phosphorylated (78.4%), with 17.7% being doubly phosphorylated and 3.9% being triply phosphorylated. The majority of phosphorylation events occurred on serine (S, 86.6%), followed by threonine (T, 13.1%) and tyrosine (Y, 0.3%). These proportions are consistent with previously reported *Arabidopsis* phosphoproteome using the same Ga^3+^-based immobilized metal affinity chromatography (Ga-IMAC) enrichment method [25]. However, they differ slightly from those reported in other tomato fruit studies [26], which showed higher Y phosphorylation (5%) using TiO_2_-based enrichment, highlighting the complementarity of different enrichment techniques. The selectivity of phosphopeptide enrichment was 97.7% at the peptide spectrum match (PSM) level and 98.3% at the peptide isoform level.

The coefficient of variation (CV) was below 0.2 in 99.3% and 96.7% of proteome and phosphoproteome datasets, respectively, confirming reproducibility. Principal component analysis (PCA) demonstrated that sample groups from different ripening stages, assessed at both the protein abundance and phosphopeptide levels, were clearly and sequentially separated, corroborating reproducibility and distinct proteomic changes during ripening (Figure 2B and C). Additionally, 43.5% of proteins identified in the proteome were phosphoproteins, and 67.9% of phosphoproteins in the phosphoproteome were quantified at the global abundance level (Figure 2D). The identified proteins and phosphoproteins were classified into functional categories, including E3 ubiquitin ligases, kinases, phosphatases, TFs, and transcription regulators (TRs) (Figure 2E). Correlation analysis between global protein abundance and RNA abundance across ripening stages [4] yielded a mean Pearson correlation coefficient of 0.44 (Figure 2F).

### Differential protein and phosphopeptide expression during ripening

Differential expression analyses were conducted between consecutive ripening stages (BR/MG, BR+2/BR, BR+4/BR+2, BR+8/BR+4) and BR+8/MG, using a fold change cutoff > 1.5 or < 0.667 and adjusted *P* < 0.01. Site-specific phosphorylation changes were normalized to corresponding protein abundance. We observed differential expression in 32.6% of proteins (*n* = 2901) and 38.3% of phosphopeptides (*n* = 11,075). Additionally, 7373 phosphopeptides showed significant site-specific changes (Table S2). Overall, protein abundance was predominantly downregulated during ripening, while site-specific phosphorylation tended to increase (Figure S1A). Differentially expressed kinases, phosphatases, TFs, TRs, and E3 ligases are shown in Table S2 and Figure S1B. Alterations at the phosphopeptide level were more significant than those at the protein level, emphasizing the regulatory importance of phosphorylation.

Gene Ontology (GO) and Kyoto Encyclopedia of Genes and Genomes (KEGG) enrichment analyses highlighted substantial biological alterations at the global protein level (Figures S2 and S3A). At the phosphopeptide level, enriched GO terms were primarily related to biological processes and molecular functions. In contrast, site-specific phosphorylation changes were enriched for cellular component terms, suggesting localization-specific phosphorylation events during ripening (Figure S4). KEGG pathways were less enriched for phosphorylation changes, reflecting the broader functional roles of protein abundance alterations (Figure S3).

**Figure 3:**
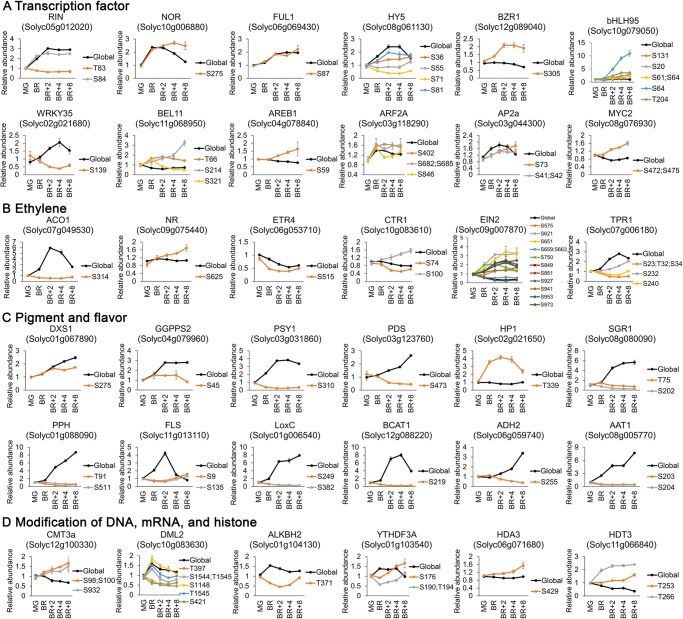
Differentially expressed p-sites of representative ripening-associated proteins The p-sites with fold changes above 1.5 or below 0.667 and adjusted *P* < 0.01 are shown. Data are represented as mean ± SD from three biological replicates. p-site, phosphorylation site; RIN, Ripening inhibitor; NOR, Non-ripening; FUL1, Fruitfull 1; HY5, Elongated hypocotyl 5; BZR1, Brassinazole resistant 1; BEL11, Bel1-like homeodomain 11; AREB1, Abscisic acid-responsive element binding protein 1; ARF2A, Auxin response factor 2A; AP2a, APETALA2a; ACO1, 1-aminocyclopropane-1-carboxylate oxidase 1; NR, never ripe; ETR4, ethylene receptor 4; CTR1, constitutive triple response 1; EIN2, ethylene-insensitive protein 2; TPR1, tetratricopeptide repeat 1; DXS1, 1-deoxy-D-xylulose-5-phosphate synthase 1; GGPPS2, geranylgeranyl pyrophosphate synthase 2; PSY1, phytoene synthase 1; PDS, phytoene desaturase; HP1, high pigment 1; SGR1, stay-green 1; PPH, pheophytinase; FLS, flavonol synthase; LoxC, lipoxygenase C; BCAT1, branched-chain aminotransferase 1; ADH2, alcohol dehydrogenase 2; AAT1, alcohol acetyltransferase 1; CMT3a, chromodomain DNA methyltransferases 3a; DML2, DEMETER-like DNA demethylases 2; ALKBH2, alpha-ketoglutarate-dependent dioxygenase homologue 2; YTHDF3A, YT521-B homology domain family protein 3a; HDA3, RPD3/HDA1 subfamily histone deacetylase 3; HDT3, HD-tuins 3; SD, standard deviation; S, serine; T, serine; Y, tyrosine.

Our data support known phosphorylation events during ripening, including reduced phosphorylation of ETR4 and increased phosphorylation of Non-ripening (NOR) upon ripening initiation [14,26] (**Figure 3**A and B; Table S2). Moreover, key ripening regulators, including Ripening inhibitor (RIN), NOR, Fruitfull 1 (FUL1), Elongated hypocotyl 5 (HY5), MYC2, and ethylene signaling components such as 1-aminocyclopropane-1-carboxylate oxidase 1 (ACO1), ETR4, NR, TPR1, Constitutive triple response 1 (CTR1), and Ethylene-insensitive protein 2 (EIN2), exhibited significant phosphorylation changes (Figure 3A and B). We identified 22 p-sites in EIN2 (Table S1), with 12 exhibiting dynamic changes, including the site S927 (corresponding to AtEIN2-S924) (Figure 3B; Table S2). The phosphopeptides containing the site T661 (corresponding to AtEIN2-T657) showed a significant decrease after the BR stage (Table S2). These findings suggest an involvement in both the classical CTR1–EIN2 and TOR-mediated pathways. Additionally, proteins involved in pigment metabolism, including 1-deoxy-D-xylulose-5-phosphate synthase 1 (DXS1), geranylgeranyl pyrophosphate synthase 2 (GGPPS2), phytoene synthase 1 (PSY1), phytoene desaturase (PDS), stay-green 1 (SGR1), high pigment 1 (HP1), and pheophytinase (PPH); and in flavor formation, such as lipoxygenase C (LoxC), alcohol dehydrogenase 2 (ADH2), alcohol acetyltransferase 1 (AAT1), and branched-chain aminotransferase 1 (BCAT1), exhibited phosphorylation changes. These changes suggest a role for phosphorylation in regulating fruit quality (Figure 3C; Table S2). Crosstalk between phosphorylation and DNA, RNA, and histone modifications was suggested by changes in p-sites of regulators, such as chromodomain DNA methyltransferases 3a (CMT3a), DEMETER-like DNA demethylases 2 (DML2), *N*^6^-methyladenosine (m^6^A) RNA demethylase ALKBH2, m^6^A reader protein YTHDF3A, and histone deacetylase HDA3 and HDT3 (Figure 3D; Table S2), warranting further investigation.

### Motif analysis of phosphoproteins during ripening

Phosphorylation motifs, which reflect kinase–substrate specificities, were analyzed using motifeR [27]. A total of 140 motifs (score > 300) were identified, including prominent motifs such as …[S/T]P… and …R…[S/T]… (Table S3), indicating activity of mitogen-activated protein kinases (MAPKs), cyclin-dependent protein kinases (CDKs), shaggy-like kinases (SLKs), SNF1-related protein kinases 2 (SnRK2s), Ca^2+^-dependent protein kinases (CPKs), Ca^2+^/calmodulin dependent protein kinases (CaMKs), and CBL-interacting protein kinases (CIPKs) [28–30]. Proline-directed motifs were predominant for T-centered p-sites, while Y sites lacked proline-directed motifs (Figure S5A), which is consistent with the reported *Arabidopsis* phosphoproteome [31]. Stage-specific motif categories, derived from upregulated phosphopeptides, revealed distinct distributions, suggesting stage-specific kinase activities and substrate relationships (Figure S5B; Table S3). These findings highlight the intricate role of phosphorylation in regulating ripening-associated processes.

### Protein co-expression network analyses at protein abundance and phosphorylation levels

**Figure 4:**
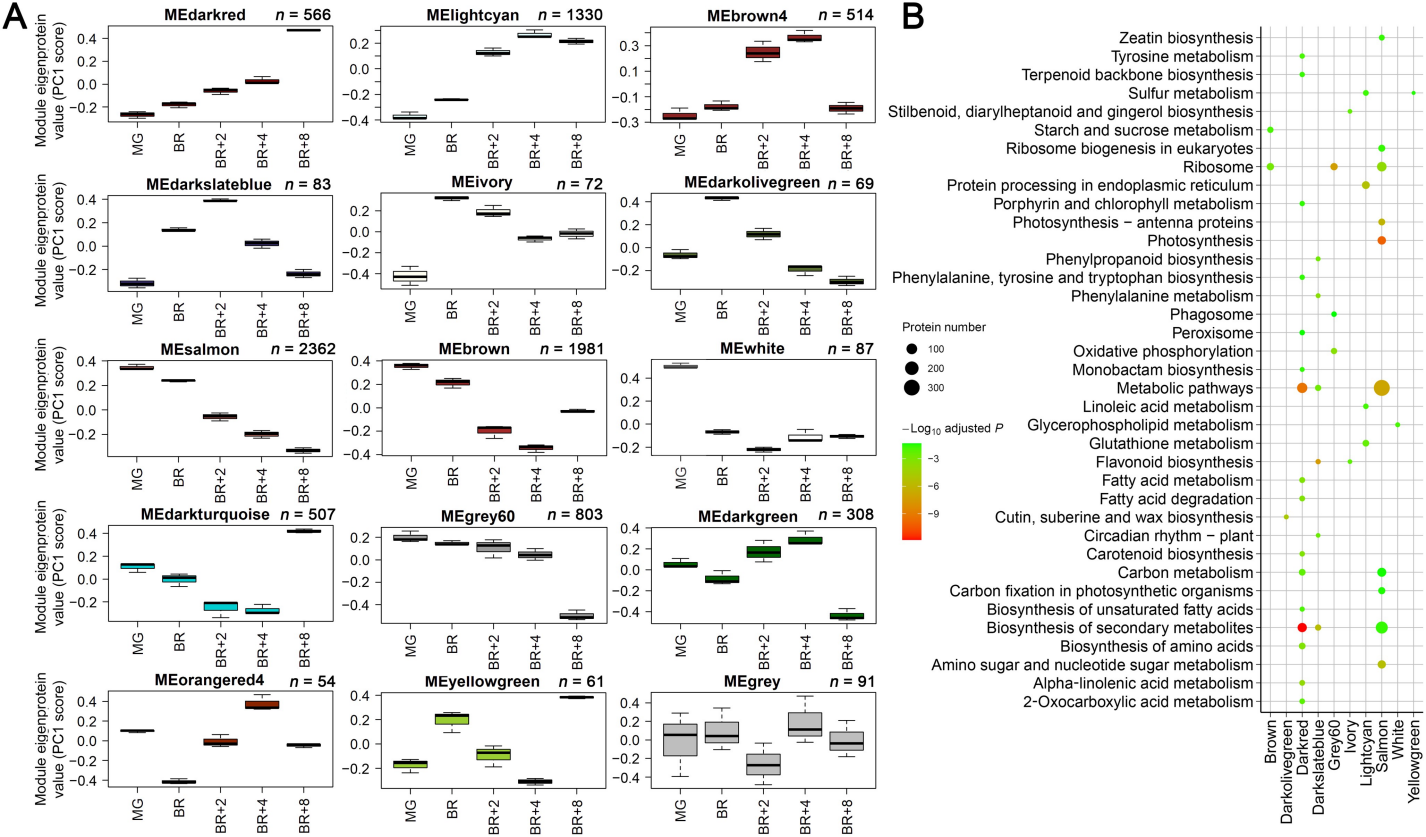
WGCNA of the AC fruit global proteome **A**. Protein co-expression modules identified via WGCNA. Boxplots display the module eigenprotein (ME) values, calculated as the first principal component of the natural log-transformed protein abundances. The number of proteins assigned to each module is indicated above the respective plot. **B**. KEGG pathway enrichment analysis of differentially expressed proteins within each module. KEGG, Kyoto Encyclopedia of Genes and Genomes; ME, module eigenprotein (equivalent to module eigengene); WGCNA, weighted gene co-expression network analysis; PC, principal component.

Co-expression network analysis, which is based on gene expression similarity, is an effective method for identifying functionally related gene networks [32]. In this study, we utilized weighted gene co-expression network analysis (WGCNA) [33,34] to analyze protein and phosphopeptide co-expression modules and examine their correlations with ripening-associated traits such as chlorophyll, β-carotene, and lycopene contents. This approach enabled us to identify major protein regulatory networks associated with the fruit ripening process. From the global proteome, we identified 15 modules, while the phosphoproteome analysis yielded 15 and 17 modules at the phosphopeptide and site-specific phosphorylation levels, respectively (**Figure 4**A, Figure S6; Table S4). KEGG pathway enrichment analysis of the differentially expressed proteins in these modules revealed significant pathway enrichment (adjusted *P* < 0.05) in the global protein modules. In contrast, only three pathways were enriched in the phosphopeptide modules, and no pathways were enriched in the site-specific phosphorylation modules (Figure 4B, Figure S7).

Correlation analysis between modules and ripening-associated traits identified several modules significantly correlated with pigment changes (chlorophyll, β-carotene, and lycopene) during ripening (Figure S8). Many previously identified ripening-regulated proteins [2,35] were assigned to these modules, underscoring WGCNA’s utility in identifying functionally significant proteins involved in fruit ripening through proteomics and phosphoproteomics datasets. For instance, the “lightcyan” module of the global proteome encompasses proteins involved in ethylene biosynthesis and signaling, such as E4, E8, ACO1, and TPR1 [36,37]; isoprenoid and carotenoid biosynthetic pathway proteins including DXS1, 4-diphosphocytidyl-2-C-methyl-D-erythritol kinase (ISPE), 2-C-methyl-D-erythritol 2,4-cyclodiphosphate synthase (IspF), GGPPS2, PSY1, and 15-*cis*-zeta-carotene isomerase (Z-ISO) [38,39]; cell wall structure-related proteins like pectate lyase (PL1), endo-1,4-beta-glucanase (Cel1 and Cel2), beta-mannanase 4a (LeMAN4a), beta-D-xylosidase (LeXYL1), and polygalacturonase (PGcat) [40–43]; and ripening-regulated TFs FUL1, Colorless non-ripening (CNR), NAM/ATAF1/2/CUC2 4 (NAC4), RIN, WRKY35, GRAS38, and others [4,44–47] (Figure 4A; Table S4). Similarly, several ripening-associated TFs, identified as phosphoproteins, were assigned to fruit pigment-correlated modules. Notable examples include RIN, CNR, NAC4, FUL1, HY5, bHLH95, WRKY17, WRKY31, ERF.D6, AREB1, and GRAS9, all of which were part of the “greenyellow” phosphopeptide module (Figure S6A; Table S4). Central hub proteins or phosphopeptides within each module were identified using two criteria: differential expression (fold change > 1.5 or < 0.667 and adjusted *P* < 0.01) and module eigengene (ME)-based gene connectivity (kME > 0.9). These hub proteins, including kinases, phosphatases, TFs, TRs, and E3 ligases, are listed in Table S5.

### REC1a: a hub protein in fruit ripening

**Figure 5:**
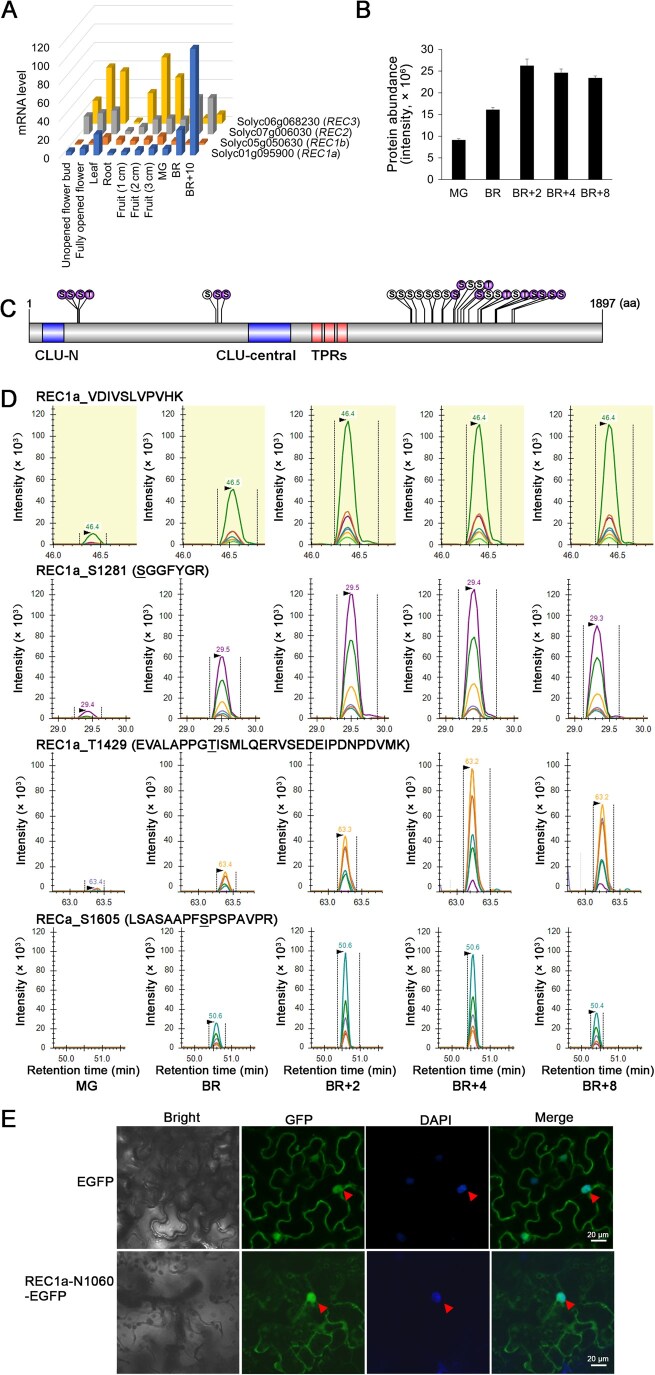
REC1a expression pattern and subcellular location **A**. Transcriptional profiles of *REC1a* and its paralogs from the Tomato eFP Browser (Rose Lab Atlas). **B**. Protein abundance of REC1a, with error bar indicating SD from three biological replicates. **C**. The p-sites identified on REC1a, with differentially expressed sites highlighted in purple. **D**. PRM validation of REC1a. Representative ion chromatograms display the abundance of REC1a and three of its p-sites, each with more than five co-eluting fragments. The p-sites of the phosphopeptides are underlined for emphasis. **E**. Confocal microscopy of *Nicotiana benthamiana* leaves transiently inﬁltrated with 35S:REC1a-N1060-EGFP or 35S:EGFP constructs, showing green EGFP fluorescence and blue DAPI nuclear staining. The red triangles indicate nuclei. Scale bar, 20 µm. REC1a, REDUCED CHLOROPLAST COVERAGE 1a; aa, amino acid; PRM, parallel reaction monitoring; GFP, green fluorescent protein; mRNA, messenger RNA; DAPI, 4’,6-diamidino-2-phenylindole; EGFP, enhanced green fluorescent protein; TPR, tetratricopeptide repeat; CLU, Clusterin.

WGCNA analysis highlighted a TPR protein (Solyc01g095900), named REC1a in this study, as a hub protein significantly associated with protein abundance, phosphopeptide, and site-specific phosphorylation levels during ripening (Table S5). REC1a, closely related to *Arabidopsis* REC1 [22], was selected for further characterization due to its central regulatory role. Its expression was predominantly observed in ripening fruits and leaves (**Figure 5**A). Protein abundance and phosphorylation levels of REC1a increased at the onset of ripening (Figure 5B; Table S2), and its phosphorylation status displayed dynamic site-specific changes during ripening (Figure 5C; Table S2). Additionally, we validated the abundance of REC1a and three of its p-sites using parallel reaction monitoring (PRM)-based targeted proteomic analysis, confirming the results from our TMT-based proteomic analysis (Figure 5D; Table S2).

To gain insights into REC1a’s molecular function, we examined its subcellular localization by transiently expressing an enhanced green fluorescent protein (EGFP)-fused REC1a fragment protein (REC1a-N1060-EGFP) in *Nicotiana benthamiana* leaves. The chimeric protein localized to the cytosol and nucleus (Figure 5E), consistent with the subcellular distribution of *Arabidopsis* REC1 [22].

**Figure 6:**
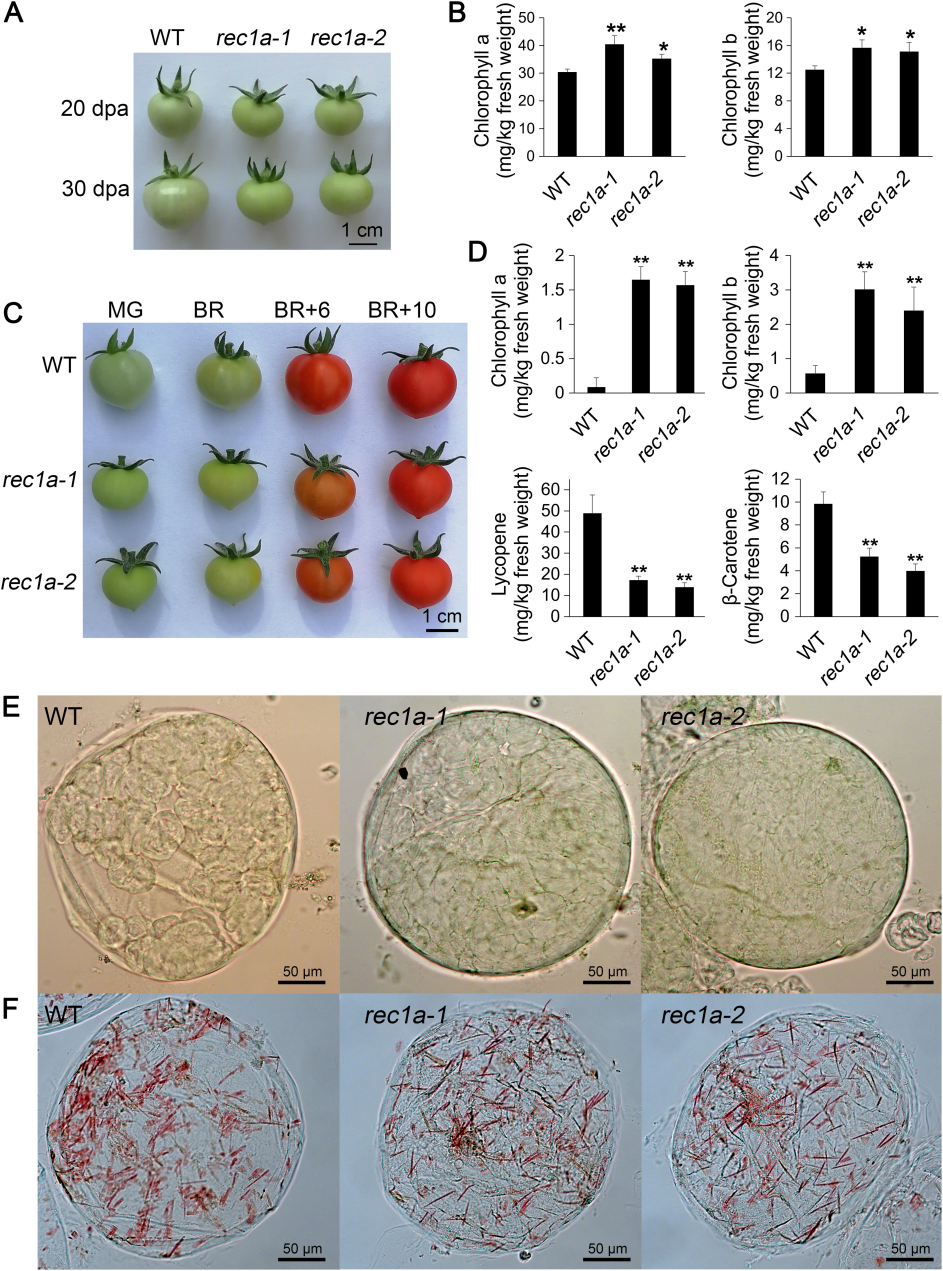
Fruit phenotype of *rec1a* mutants **A**. Immature fruits. **B**. Chlorophyll content in fruits at 20 dpa. **C**. Fruits at various ripening stages. **D**. Chlorophyll, lycopene, and β-carotene contents at the BR+10 stage. **E**. and **F**. Representative pericarp cells at 25 dpa (E) and BR+10 (F), isolated from the equator region. In (B and D), data are represented as mean ± SD of four biological replicates. Statistical significance was determined via Student’s *t*-test (*, *P* < 0.05; **, *P* < 0.01). WT, wild-type; dpa, days post anthesis; BR+6, breaker + 6 days; BR+10, breaker + 10 days.

To investigate REC1a’s role in fruit ripening, two CRISPR-associated protein 9 (Cas9)-free homozygous mutant lines (*rec1a-1* and *rec1a-2*) were generated using clustered regularly interspaced short palindromic repeats (CRISPR)-Cas9-mediated gene editing in the Micro-Tom cultivar, a model system chosen for its rapid life cycle and small size. No off-target effects were detected in the mutant lines (Figure S9). Loss of *REC1a* function led to reduced seed germination, an increased proportion of three-cotyledon seedlings, delayed flowering, and a shorter fruit development period (from anthesis to the BR stage) (Figure S10A–E). Immature *rec1a* mutant fruits displayed deeper coloration and higher chlorophyll levels than the wild-type (**Figure 6**A and B). At the red-ripe stage, mutant fruits exhibited reduced β-carotene and lycopene accumulation and retained trace amounts of chlorophyll, which were higher than those in wild-type fruits (Figure 6C and D, Figure S10F). Additionally, mutant fruits were smaller and had increased total soluble solids compared to wild-type fruits (Figure S10G and H). In mutant leaves, chlorophyll content was lower, mirroring the phenotype observed in *Arabidopsis rec1* mutants [22] (Figure S11A).

To further characterize plastid morphology, including chloroplasts and chromoplasts, we analyzed their distribution using glutaraldehyde-fixed leaf and fruit pericarp cells. In line with *Arabidopsis rec1* mutants, *rec1a* mutant mesophyll cells showed reduced chloroplast coverage (Figure S11B and C). Additionally, increased cell size, reduced chloroplast plan area, and no significant change in chloroplast number per cell were observed in *rec1a* mutant mesophyll cells (Figure S11D–F). In tomato fruit pericarp, the cell size, plastid size, and plastid number exhibit significant variation [48]. Chloroplasts were densely packed in immature *rec1a* mutant fruit pericarp cells [25 days post anthesis (dpa)], in contrast to the loosely distributed chloroplasts observed in wild-type cells (Figure 6E). This packing likely accounts for the increased chlorophyll content in *rec1a* mutant fruits (Figure 6A and B). In addition to variations in cell size, plastid size, and plastid number, chromoplasts with distinct pigment sequestration substructures coexist in ripe tomato fruits [38]. In mature fruits, the chromoplasts of *rec1a* mutants had a reduced coverage area of lycopene sequestration substructures, which likely contributed to decreased lycopene accumulation (Figure 6C, D, and F).

### Quantitative proteomic analysis reveals protein alterations in *rec1a* mutants

A TMT-based quantitative proteomic analysis was conducted to investigate protein alterations in fruits with a loss of *REC1a* function. The experimental workflow is illustrated in Figure S12. CV analysis demonstrated high reproducibility among biological replicates (Figure S13A). PCA revealed distinct clustering of sample groups, including internal references, highlighting significant proteomic differences between mutants and wild-type fruits across ripening stages (Figure S13B and C). From a total of 9407 identified protein groups (FDR < 1%), 8355 were quantified in all samples (Table S6). Of these, 1047 proteins exhibited significant differential expression at the MG, BR, or breaker + 6 days (BR+6) stages (fold change > 1.5 or < 0.667 and adjusted *P* < 0.01) (Table S7). GO and KEGG enrichment analyses of these differentially expressed proteins revealed ripening stage-specific enrichment patterns: terms associated with carbohydrate metabolism were predominant at the MG stage, whereas terms related to plastid organization were notably prominent at the BR+6 stage (Figure S14A–D). These results suggest that REC1a influences both carbohydrate metabolism and plastid functions, aligning with observed mutant phenotypes (Figure 6, Figure S10). Additionally, pathway analysis highlighted disruptions in secondary metabolism pathways in the mutants (Figure S14D).

**Figure 7:**
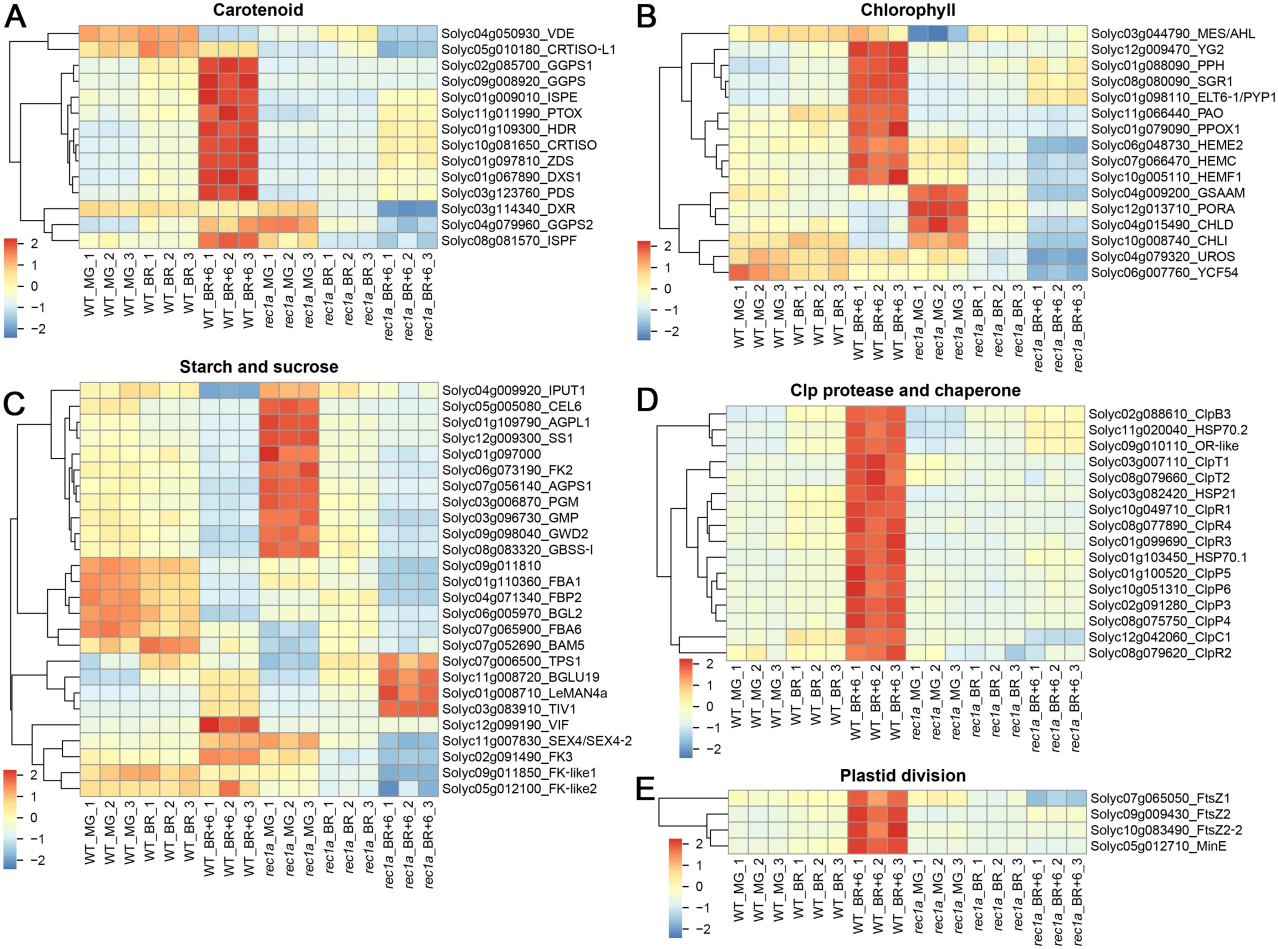
Expression patterns of representative proteins associated with the *rec1a* mutant phenotype VDE, violaxanthin de-epoxidase; GGPPS, geranylgeranyl pyrophosphate synthase; DXS1, 1-deoxy-D-xylulose-5-phosphate synthase 1; SSU-II, GPP synthase small subunit type II; DXR, 1-deoxy-D-xylulose-5-phosphate reductoisomerase; CRTISO, carotene *cis*-trans isomerase; PDS, phytoene desaturase; HDR, 1-hydroxy-2-methyl-2-(E)-butenyl 4-diphosphate reductase; ISPE, 4-diphosphocytidyl-2-C-methyl-D-erythritol kinase; ZDS, zeta-carotene desaturase; IspF, 2-C-methyl-D-erythritol 2,4-cyclodiphosphate synthase; PTOX, plastid terminal oxidase; PORA, protochlorophyllide oxidoreductase A; MES, methylesterase; CHLD, magnesium chelatase subunit D; CHLI, magnesium chelatase subunit I; PPH, pheophytinase; PAO, pheophorbide a oxygenase; HEMC, hydroxymethylbilane synthase; HEME2, uroporphyrinogen decarboxylase; HEMF1, coproporphyrinogen III oxidase; SGR1, stay-green 1; GSAM, glutamate-1-semialdehyde 2,1-aminomutase; YG2, yellow-green 2; PPO1, protoporphyrinogen IX oxidase 1; UROS, uroporphyrinogen III (Uro) synthase; PYP1, pale yellow petal 1; YCF54, hypothetical chloroplast open reading frame 54; AGPL1, ADP-glucose pyrophosphorylase (AGP) large subunit 1; AGPS1, AGP small subunit 1; SS1, sucrose synthase 1; FBP2, fructose-1,6-bisphosphatase 2; FK2, fructokinase 2; FK3, fructokinase 3; FK-like, fructokinase-like protein; FBA6, fructose-1,6-bisphosphate aldolase 6; PGM, phosphoglucomutase; BGL2, beta-glucosidase 2; GWD2, glucan water dikinase 2; CEL6, CELLULASE 6; GBSS-I, granule-bound starch synthase I; BAM5, beta-amylase 5; GMP, GDP-D-mannose pyrophosphorylase; FBA1, fructose-1,6-bisphosphate aldolase 1; LeMAN4a, beta-mannanase 4a; SEX4, starch excess 4; BGLU19, beta-glucosidase 19; IPUT1, inositol phosphorylceramide glucuronosyltransferase 1; TIV1, acid invertase; VIF, vacuolar invertase inhibitor; TPS1, trehalose-6-phosphate synthase 1; HSP, heat shock protein; OR-like, orange like; Clp, caseinolytic protease; FtsZ, filamenting temperature-sensitive Z; MinE, minicell locus E.

To explore the molecular basis of phenotypic changes in the mutants, we examined proteins involved in pigment and sugar metabolism. Consistent with reduced lycopene and β-carotene levels in ripe mutant fruits, proteins involved in carotenoid biosynthesis were markedly downregulated at the BR and BR+6 stages (**Figure 7**A). Photosynthesis in green tomato fruit contributes up to 20% of photosynthate within the fruit [49]. Consequently, the chlorophyll and carotenoid content of the fruit is crucial for sugar production and nutritional formation in tomato fruits. Proteins associated with chlorophyll degradation, such as methylesterase (MES) (MG stage), SGR1, pale yellow petal 1 (PYP1), pheophorbide an oxygenase (PAO), and PPH (BR+6 stage), were less abundant in mutants. Conversely, proteins involved in chlorophyll biosynthesis, including glutamate-1-semialdehyde 2,1-aminomutase (GSAM), magnesium chelatase subunit D (CHLD), magnesium chelatase subunit I (CHLI), and protochlorophyllide oxidoreductase A (PORA), showed higher levels at the MG stage (Figure 7B), correlating with higher chlorophyll levels in mutant fruits (Figure 6B and D). Proteins related to starch biosynthesis, such as granule-bound starch synthase I (GBSS-I), phosphoglucomutase (PGM), and adenosine diphosphate (ADP)-glucose pyrophosphorylase subunits (AGPS1 and AGPL1), were elevated at the MG stage (Figure 7C), supporting increased total soluble solids content in ripe mutant fruits (Figure S10H). REC1a mutation significantly affected plastid development. Most subunits of the Clp protease complex and associated chaperones, crucial for plastidial protein quality control and chloroplast–chromoplast differentiation [50,51], were downregulated in mutants, especially at the BR and BR+6 stages (Figure 7D). Additionally, plastid division proteins such as filamenting temperature-sensitive Z1 (FtsZ1), FtsZ2, and minicell locus E (MinE) [52] were significantly decreased in ripe mutant fruits (Figure 7E), implicating REC1a in plastid division and protein homeostasis.

### Transcriptional expression patterns in *rec1a* mutants

To understand proteomic changes in *rec1a* fruits, we analyzed transcript levels of proteins implicated in key phenotypic processes. Most showed parallel mRNA and protein expression trends during ripening, except for GGPPS2 and MinE (Figure S15). GGPPS is critical for catalyzing the synthesis of geranylgeranyl diphosphate (GGPP), which is the common precursor for most photosynthesis-related isoprenoids, including chlorophyll and carotenoids [53]. While the transcript levels of *GGPPS2* in the *rec1a* mutant were elevated, its protein levels decreased during ripening, which contrasts with the increased expression observed in the wild-type at both the transcriptional and protein levels (Figure S15). Similarly, MinE transcript levels increased during ripening, but protein levels remained unchanged (Figure S15). These results suggest that proteomic alterations in *rec1a* mutants are largely driven by transcriptional regulation, with potential post-transcriptional control of specific genes like *GGPPS2* and *MinE* influencing pigment accumulation and plastid development. Further transcriptional analysis of unquantified TFs and E3 ligases related to plastid development and pigment accumulation revealed differential expression patterns in the mutants. TFs such as BL4, NAC4, MYB72, and the class I knotted1-like homeobox TFs TKN2 and TKN4, displayed altered expression compared to wild-type fruits (Figure S16), reinforcing the role of REC1a in regulating ripening-associated processes.

### Co-expression network analysis of *rec1a* mutants

**Figure 8:**
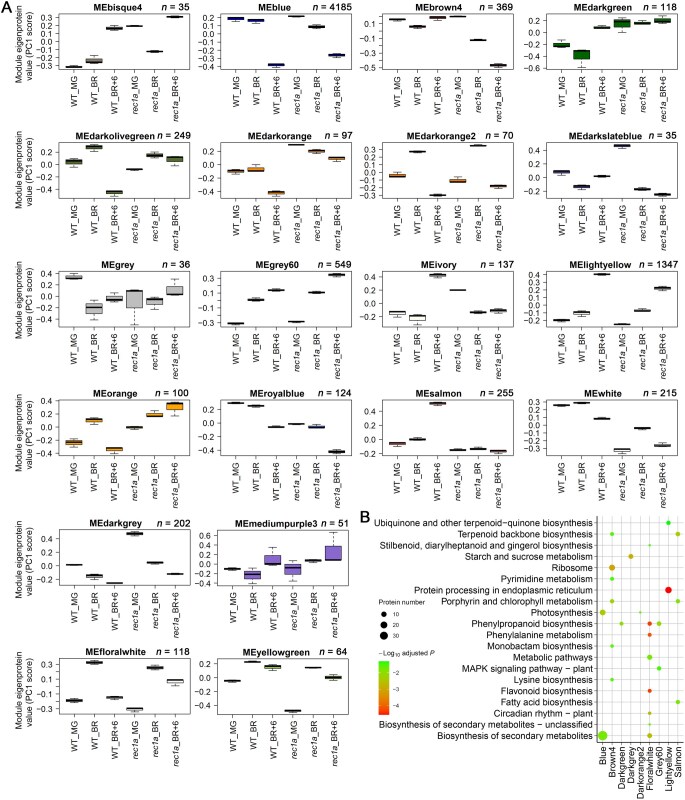
WGCNA of the proteomes from *rec1a* mutant and WT fruits **A**. Identified protein co-expression modules. Boxplots display the ME values, representing the first principal component of the natural log-transformed protein abundances. Protein counts for each module are shown above the corresponding plots. **B**. KEGG pathway enrichment analysis of the differentially expressed proteins within each module.

WGCNA analysis identified 20 protein modules with differential expression patterns (**Figure 8**A). Six modules showed strong correlations with fruit pigment contents (Pearson correlation coefficient > 0.8 or < −0.8 and adjusted *P* < 0.05) (Figure S17). Enrichment analysis revealed module-specific biological pathways. For instance, the “salmon” module, enriched in “terpenoid backbone biosynthesis” and “porphyrin and chlorophyll metabolism” pathways, was closely associated with the mutant phenotype (Figure 8B, Figure S17). Within this module, proteins involved in carotenoid biosynthesis, plastid division, and Clp protease complexes were notably downregulated in mutants (Figure 8A; Table S8). Hub proteins (kME > 0.8) within modules included those implicated in pigment, flavonoid, starch, and melatonin biosynthesis or degradation, as well as ethylene signaling and cell wall structure (Table S8). Additionally, 33 hub proteins belonging to kinases, phosphatases, TFs, TRs, and E3 ligases were uncovered, providing valuable insights into the molecular mechanisms of REC1a-mediated ripening regulation.

### REC1a interacts with eukaryotic translation initiation factors

**Figure 9:**
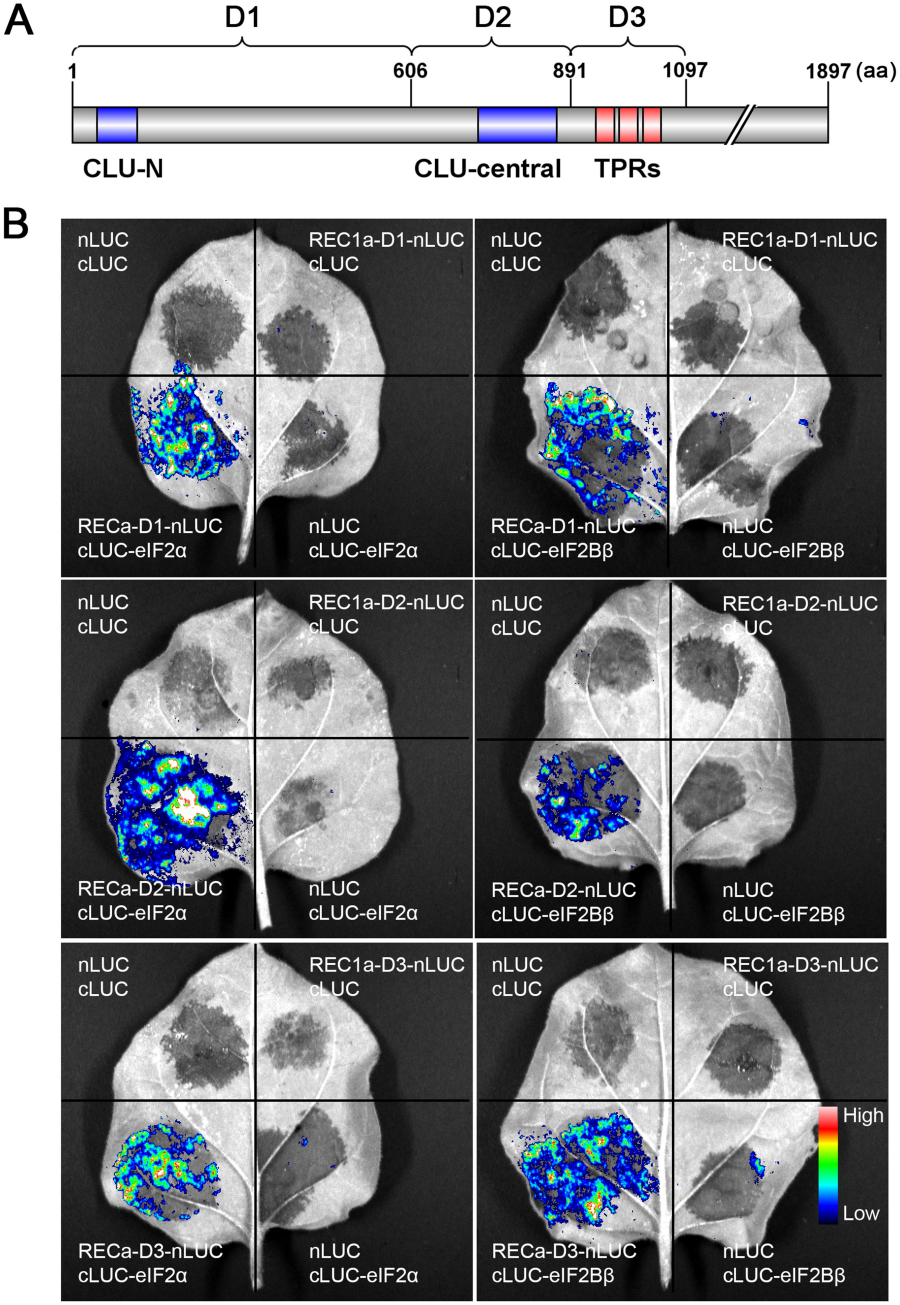
REC1a interacts with eukaryotic translation initiation factor subunits eIF2**α** and eIF2B**β** **A**. Diagram of REC1a fragments (D1, D2, and D3) used in split-luciferase complementation assays. **B**. Luciferase signals detected in *Nicotiana benthamiana* leaves, indicating interactions.

To further understand how REC1a regulates fruit ripening, we performed co-immunoprecipitation (Co-IP) analysis to isolate REC1a-interacting proteins using anti-green fluorescent protein (GFP) antibodies from immature tomato fruits (25 dpa) overexpressing REC1a-N1060-EGFP, with fruits overexpressing EGFP at the same stage as a negative control. Mass spectrometry (MS) analysis of the captured protein complexes identified several interacting candidates, including subunits of the eukaryotic translation initiation factor complexes eIF2 and eIF2B, which were highly expressed in the overexpressing samples but undetectable in the negative control samples (Table S9). We employed a split-luciferase complementation assay to verify the molecular interactions between REC1a and two selected candidates, the eukaryotic translation initiation factor subunits eIF2α and eIF2Bβ. Interestingly, we found that three fragments of REC1a (D1, D2, and D3), each containing a distinct domain [Clusterin (CLU)-N, CLU-central, and TPR repeats], interacted with both eIF2α and eIF2Bβ (**Figure 9**). These findings suggest that REC1a may regulate fruit ripening and plastid development by modulating translation processes, as eukaryotic translation initiation factors are known to influence ribosome assembly and specific mRNA translation [54–56]. Additionally, no interactions were observed between the three REC1a fragments and their paralogs (REC1b, REC2, and REC3), indicating functional specificity.

## Discussion

In this study, we identified 11,691 unique proteins, encompassing both global proteins and phosphoproteins, from tomato fruit pericarps of the Ailsa Craig and Micro-Tom varieties (Figure S18A). These proteins account for 33.7% of the protein-coding genes annotated in the tomato genome (ITAG2.4, with 34,725 entries). Compared with a comprehensive transcriptome analysis [4], our findings provided protein-coding evidence for 53.6% of the pericarp transcripts, with over 300 proteins uniquely identified in our proteomic dataset that were absent from the transcriptome data (Figure S18A). Moreover, we expanded the phosphoproteome by identifying over 20,000 high-confidence p-sites in ripening tomato fruits, 67.7% of which were novel compared to previous studies on tomato leaf phosphoproteomes [57] (Figure S18B). This work represents the most extensive proteome and phosphoproteome datasets for ripening tomato fruits to date. However, the proteomic coverage of 33.7% remains below the transcriptomic coverage of 71% for coding genes in tomato fruits [4].

The correlation between protein abundance and transcript levels varies across tissues and developmental stages in plants [58,59]. Our study revealed a relatively strong positive correlation between protein and transcript expression patterns during ripening, consistent with transcriptome data from similar ripening stages [4] (Figure 2F). Nevertheless, discrepancies were observed. A subset of differentially expressed proteins (*n* = 98) showed no significant transcript-level changes, while others exhibited negative correlations with their transcripts (55 proteins, Pearson correlation coefficient < −0.5, adjusted *P* < 0.05) (Figure S18C and D). These results suggest that post-transcriptional, translational, and post-translational regulation significantly influence ripening, while differences in sample sources may also contribute to the discrepancies.

We observed that 43.5% of the globally identified proteins carried p-sites, consistent with previous *Arabidopsis* studies, where 47% of the proteome contained p-sites [31]. Most phosphoproteins (87%) exhibited less than 10% of their S/T/Y residues phosphorylated (Figure S19A). However, certain protein categories, such as serine/arginine-rich (SR) proteins involved in alternative splicing [60], showed higher phosphorylation densities. Among these, 11 SR and SR-like proteins displayed over 20% S/T/Y phosphosite occupancy, with significant phosphorylation changes observed during ripening (Figure S19B; Table S2). These findings underscore the importance of phosphorylation in regulating alternative splicing during fruit ripening. Additionally, we identified over 120 E3 ligases with significant phosphopeptide-level changes, highlighting intricate crosstalk between phosphorylation and ubiquitination in fruit ripening.

Through WGCNA-based proteome and phosphoproteome analysis, we identified the TPR protein REC1a as a key regulator of fruit development and ripening. Functional characterization of REC1a confirmed its role in promoting leaf chlorophyll content, consistent with its *Arabidopsis* homolog [22]. Furthermore, REC1a emerged as a positive regulator of lycopene accumulation and chromoplast development in ripening fruits, aligning with the homologous function of REC2/RCP2 in promoting carotenoid biosynthesis and chromoplast differentiation in monkeyflower floral tissues [23]. Notably, while immature tomato fruits with larger chloroplast compartments and higher chlorophyll content generally accumulate more carotenoids during ripening [48,61–63], the *rec1a* mutants exhibited dense chloroplast packing and increased chlorophyll content in immature fruits but ultimately accumulated less lycopene and β-carotene in ripe fruits. These findings suggest that REC1a is a critical regulator of chloroplast-to-chromoplast transition during ripening. Additionally, the reduced accumulation of plastidial Clp protease complex proteins in *rec1a* mutants after the BR stage reinforces REC1a’s role in facilitating this plastid transition, as Clp proteases are essential for plastidial protein quality control and chromoplast differentiation [50,51].

While REC1a positively regulates chloroplast compartment size and chlorophyll content in leaves, the increased chlorophyll content in *rec1a* mutant fruits presents a functional divergence. Functional redundancy among REC paralogs may explain this discrepancy, as *Arabidopsis* RECs are known to exhibit overlapping functions [22]. In tomato, *REC1a* has three paralogs: *REC1b*, *REC2*, and *REC3*. While *REC1b* expression is low in tomato fruits, *REC2* and *REC3* exhibit higher expression during ripening and in immature fruits, respectively (Figure 5A). Our proteomic analysis showed REC3 upregulation in *rec1a* mutants (Figure S20A). Transcript analysis revealed that *REC1b* expression decreased slightly, *REC2* remained unchanged, and *REC3* was significantly upregulated at the 20 dpa and MG stages (Figure S20B). These results suggest that *REC3* may compensate for *REC1a* loss in regulating chlorophyll biosynthesis and plastid development in tomato fruits.

Finally, we demonstrated that REC1a interacts with subunits of the eukaryotic translation initiation factor complexes eIF2 and eIF2B. Because translation initiation factors form distinct ribosomal subpopulations to facilitate gene-specific translation [54,64], we propose that REC1a modulates translation initiation to regulate ripening-associated protein synthesis. Future studies should identify REC1a-targeted mRNAs and elucidate the mechanisms by which REC1a and its interactors control their translation to mediate fruit ripening and plastid development.

## Materials and methods

### Plant material and fruit sampling

Tomato seeds (cultivars Ailsa Craig and Micro-Tom) were surface-sterilized with 5% sodium hypochlorite (w/v) for 20 min, thoroughly rinsed six times with sterilized water, and sown in peat-based substrate mix (LM-2 Germination Mix, Lambert, Quebec, Canada). Plants were grown in a greenhouse under controlled conditions: 24°C, a 12 h light/12 h dark cycle, 75% relative humidity, and 250 μmol/m^2^·s light intensity. Fruit pericarp samples from Ailsa Craig were collected at MG (approximately 39 dpa), BR, BR+2, BR+4, and BR+8 stages for global proteome and phosphoproteome analyses. Fruit pericarp samples from Micro-Tom and the *rec1a* mutant were collected at MG, BR, and BR+6 stages for proteome quantification. All samples were snap-frozen in liquid nitrogen, ground into a fine powder, and stored at −80°C.

### Protein isolation, digestion, and TMT labeling

Proteins were extracted under denaturing conditions using a modified phenol-methanol protocol [65]. Following the method outlined in [25], proteins were reduced with tris(2-carboxyethyl)phosphine hydrochloride (TCEP), alkylated with iodoacetamide, and precipitated by mixing with five volumes of cold acetone. The mixture was incubated at −40°C overnight. The protein pellet was washed sequentially with cold acetone:methanol:water (12:1:1.4, v/v) and cold methanol, and then dissolved in 50 mM triethylammonium bicarbonate (TEAB). Proteins were digested with trypsin at an enzyme:substrate ratio of 1:25 for 16 h at 37°C.

For TMT labeling, digested peptides (220 μg) from 15 Ailsa Craig samples (three biological replicates for MG, BR, BR+2, BR+4, and BR+8) were labeled with 15 reagents from a TMTpro 16-plex set (Catalog No. A44521, Thermo Fisher Scientific, Waltham, MA) (Figure 1). For the *rec1a* mutant and Micro-Tom analyses, peptides (20 μg) from 11 samples (three biological replicates for MG, BR, and BR+6, and two pooled internal reference samples) were labeled with a TMT10-plex set (Catalog No. 90110, Thermo Fisher Scientific, Waltham, MA) plus an additional 131C label (A34807, Thermo Fisher Scientific) (Figure S12). Internal reference samples were mixtures of all 18 peptide samples from the mutant and wild-type fruits. The labeling reaction was terminated by adding hydroxylamine to 1% (final concentration) and incubating at room temperature for 15 min.

### Peptide fractionation and phosphopeptide enrichment

Labeled peptides from Ailsa Craig were pooled, and approximately 250 μg were fractionated into 31 fractions and further combined into 13 pools for global proteome analysis. The remaining 3050 μg were fractionated into 31 fractions and combined into seven pools for phosphoproteome enrichment (Figure 1). Similarly, labeled peptides from the *rec1a* mutant and wild-type were fractionated into 31 fractions, combined into 14 pools each for proteome analysis (Figure S12). Fractionation was conducted using an ACQUITY UPLC H-Class system (Waters, Milford, MA) with a Waters ACQUITY UPLC BEH C18 Column (4.6 mm × 250 mm, 3.5 μm particle size) at 600 μl/min. Peptides were separated by a gradient starting from 2% phase B [100% acetonitrile (ACN), 5 mM ammonium hydroxide] for 5 min, increasing to 4% buffer B in 1.0 min, followed by 4%–36% buffer B for 31.0 min, 36%–70% buffer B for 4.6 min, and 70% phase B for 9.4 min. Phase A was 5 mM ammonium hydroxide in water. Fractions were dried with a SpeedVac (Labconco, Kansas, MO). For liquid chromatography with tandem mass spectrometry (LC-MS/MS) analysis, peptides for global proteome quantification were reconstituted in 0.5% trifluoroacetic acid (TFA).

Phosphopeptides were enriched using Ga-IMAC [25]. Sepharose slurry (GE HealthCare, Piscataway, NJ) was charged with GaCl_3_ and incubated with peptides in 80% ACN in 0.1% TFA for 45 min. After washing, phosphopeptides were eluted with 50% ACN in 5% ammonium hydroxide, desalted with home-made C18 Stage Tips, vacuum-dried, and reconstituted in 0.1% TFA for LC-MS/MS.

### MS analysis

Peptides were analyzed using an UltiMate 3000 RSLCnano UHPLC system (Thermo Fisher Scientific) coupled to a Q Exactive HF Hybrid mass spectrometer (Thermo Fisher Scientific, Waltham, MA). The global proteome samples were injected three times, while the phosphoproteome samples were injected twice to improve identification coverage.

Peptides were trapped on a C18 column (PepMap, 100 Å, 100 μm × 2 cm, 5 μm) and separated on an analytical column (PepMap, 100 Å, 75 μm × 50 cm, 2 μm). A binary gradient of 0.1% formic acid (phase A) and 80% ACN in 0.1% formic acid (phase B) was applied. For global proteome peptides, samples were equilibrated in 5% buffer B and separated using a gradient elution: 5%–8% buffer B over 2 min, 8%–20% buffer B over 66 min, and 20%–40% buffer B over 33 min, followed by a final increase to 90% buffer B within 4 min. Enriched phosphopeptides were equilibrated in 5% buffer B and eluted with a gradient: 8%–11% buffer B within 5 min, 11%–43% buffer B within 67 min, and a final increase to 90% buffer B within 6 min.

The full MS scan (*m/z* 350–1650) was acquired at a resolution of 120,000 (*m/z* 200), with an automatic gain control (AGC) target of 3 × 10^6^. MS/MS scans were acquired in top-12 mode with a resolution of 45,000 (*m/z* 200) and a normalized collision energy of 32%. Other settings included charge state exclusion for unassigned, +1, and > 6 ions, and a 30 s dynamic exclusion window.

### MS data and bioinformatics analysis

Raw data were processed in Proteome Discoverer (v2.4.0.305) using SEQUEST HT and MS Amanda search engines against the ITAG2.4 tomato protein database (34,725 entries). The searches were processed with a 10 ppm precursor ion mass tolerance and 0.02 Da fragment mass tolerance for SEQUEST HT, and 5 ppm MS1 tolerance and 0.02 Da MS2 tolerance for MS Amanda. Other search parameters included trypsin specificity (max two missed cleavages), static modifications for carbamidomethylation and TMTpro tags, dynamic modifications for methionine oxidation and *N*-terminal acetylation, and a maximum of three equal modifications per peptide. For phosphopeptides, phosphorylation (+79.966 Da) was included as an additional dynamic modification on S, T, and Y residues, and PhosphoRS mode was activated in ptmRS node. A reversed sequence decoy strategy was used for peptide false discovery estimation, and Percolator was used to validate identifications. The identified PSMs, peptides, and proteins were filtered at 1% FDR level (*q* scores < 0.01). To account for slight sample loading and labeling efficiency variation, normalization was applied for the grand total reporter ion intensity for each channel of the 15-plex experiment. In addition, for the quantitative comparison of *rec1a-1* and wild-type, internal reference scaling normalization [66] was performed using the two pooled reference channels to correct the random sampling between the TMT experiments.

Statistical analyses and visualization (*e.g.*, PCA, violin plots, and heatmaps) were performed using R packages, including limma, ggplot2, and pheatmap. Functional annotations were performed with eggNOG-mapper, TBtools, and KEGG Orthology Based Annotation System (KOBAS 3.0). Co-expression analyses used the WGCNA package (v1.70-3) in R, with parameters adjusted for global and phosphoproteome datasets.

### PRM

Three biological replicates were performed at each ripening stage for PRM validation. Both phosphorylated and non-phosphorylated peptides were analyzed using PRM assays as described previously [25], with minor adjustments. Briefly, a full MS scan (330–1500 m/z) was conducted at a resolution of 60,000 at m/z 200, with an AGC target of 3 × 10^6^ ions and a maximum injection time of 100 ms. This was followed by scheduled and targeted MS/MS scans at a resolution of 30,000 at m/z 200, with an AGC target of 1 × 10^5^ ions, a maximum injection time of 50 ms, and a collision energy of 27%.

Raw data from the PRM validation were analyzed using Skyline (v22.2.0.312) with manual checks, including assessments of peak shape and retention time. Selected precursors met the following criteria: a library dot product (dotp) > 0.8, an isotope dot product (idotp) > 0.8, mass error below 10 ppm for precursors, and 20 ppm for fragment ions. For p-site localization, up to 35 ions identified in the MS/MS spectra were manually inspected. Two HSP70 proteins (Solyc03g082920 and Solyc11g066060) were used as internal references for normalizing the loading of the global protein samples, due to their low variation across all samples, as observed in previous TMT-based assays. Total phosphopeptide intensity from the data-dependent acquisition (DDA) assay of each sample was used for phosphopeptide loading. The peptides and isolation lists used for validating proteins or phosphopeptides are detailed in Table S10.

### Plasmid construction and transformation

To generate the 35S:REC1a-N1060-EGFP construct, the *N*-terminal *REC1a* fragment (1060 amino acids) was cloned in-frame with *EGFP* and inserted into pCAMBIA1301. A 35S:EGFP plasmid was constructed as a control. Single-guide RNAs (sgRNAs) targeting *REC1a* were designed using CRISPR-P 2.0 [67] and assembled into pYLCRISPR/Cas9P_ubi_-H, which was kindly provided by Dr. Yao-Guang Liu of South China Agricultural University. All constructs were introduced into *Agrobacterium tumefaciens* GV3101 and transformed into tomato via cotyledon puncture method [68]. Potential off-target sites were assessed using CRISPR-P 2.0 and subsequently validated with Sanger sequencing. The sgRNAs and primers used for vector construction and mutation confirmation are listed in Table S10.

### Pigment and total soluble solids analyses

Chlorophyll and carotenoid pigments were extracted with acetone:hexane (2:3, v/v) and quantified spectrophotometrically using established equations [69]. Total soluble solids (°Bx) were measured with a digital refractometer (PAL-1, ATAGO, Tokyo, Japan).

### Microscopy and subcellular localization

Cell isolation and plastid morphology characterization were conducted as described previously [70], with slight adjustments. Briefly, leaf (4-week-old) or pericarp tissues were fixed in 3.5% (v/v) glutaraldehyde for 1 h in darkness immediately after being excised into 1–2 mm^2^ sections with a razor blade. The leaf and immature pericarp tissues were disrupted by heat treatment at 65°C in 0.1 M ethylenediaminetetraacetic acid disodium (EDTA-Na_2_) solution (pH 9.0) for 4 h and 30 min, respectively, while the red ripe fruit pericarp was treated at room temperature for 30 min. Samples were mounted on microscope slides and observed using a ZEISS Axio Vert.A1 Microscope (Carl Zeiss AG, Oberkochen, Germany). The cell or plastid plan area was measured using ImageJ software (v5.1). Chloroplast coverage in mesophyll cells (25 cells per genotype) was calculated as: (chloroplast number per cell × mean chloroplast plan area per cell) / mesophyll cell plan area, following the method described previously [70].

For subcellular location analysis, the 35S:REC1a-N1060-EGFP and 35S:EGFP vectors were introduced into *A. tumefaciens* strain GV3101, followed by infiltration into *N. benthamiana* leaves. The fluorescent proteins were expressed for 48 h before being captured using a confocal laser scanning microscope (TCS-SP5, Leica, Wetzlar, Germany). 4’,6-diamidino-2-phenylindole (DAPI) staining of nuclei was performed for 1 h prior to fluorescence observation.

### The Co-IP analysis

Pericarps of immature fruits (25 dpa) were ground into powder using liquid nitrogen and resuspended with lysis buffer [20 mM N-(2-Hydroxyethyl)piperazine-N′-(2-ethanesulfonic acid) (HEPES) (pH 7.5), 5% glycerol, 100 mM NaCl, 5 mM MgCl_2_, 1% Triton X-100, 1 mM phenylmethylsulfonyl fluoride (PMSF)] containing protease and phosphatase inhibitor cocktails for protein extraction. The protein extracts were incubated with nProtein A Sepharose 4 Fast Flow (GE HealthCare, Uppsala, Sweden) covalently immobilized with anti-GFP polyclonal antibodies for 2 h at 4°C. The beads were washed three times with lysis buffer, followed by two additional washes with 20 mM Tris-HCl (pH 8.0) containing 2 mM CaCl_2_. After removing residual liquid, the beads were resuspended in 20 mM Tris-HCl (pH 8.0) and sequentially incubated with 4 mM dithiothreitol (DTT) for 30 min at room temperature and then with 6 mM iodoacetamide for 10 min. Trypsin digestion was performed at a 1:50 (w/w) trypsin-to-protein ratio by incubating the mixture at 37°C overnight. The reaction was halted by adding formic acid to a final concentration of 1% (v/v). The digested peptides were desalted using StageTips [71] and analyzed via LC-MS/MS following the global proteome analysis method described above, with adjusted chromatographic and spectral acquisition parameters. Briefly, the elution gradient included the following steps: 5%–8% buffer B over 1.5 min, 8%–40% buffer B over 96.5 min, and a final ramp to 90% buffer B over 4 min. MS/MS spectra were acquired in data-dependent top-15 mode using a resolution of 30,000 and a normalized collision energy of 27%. Raw data files were searched with a preset LFQ-MBR workflow in FragPipe 22.0 with MSFragger (v4.1) [72], against the tomato ITAG2.4 proteome.

### Split-luciferase complementation assay

Three fragments of *REC1a*, namely D1 (amino acids 1–606, containing the CLU-N domain), D2 (amino acids 607–891, containing the CLU-central domain), and D3 (amino acids 892–1097, containing the TPR repeats domain), were separately ligated into the pCAMBIA1300-nLUC vector. Meanwhile, the full-length coding sequences of *eIF2α*, *eIF2Bβ*, *REC1b*, *REC2*, and *REC3* were cloned into the pCAMBIA1300-cLUC vector. These constructs were transformed into *A. tumefaciens* GV3101 and transiently expressed in *N. benthamiana* leaves for two days. D-luciferin (1 mM) was infiltrated into the leaves and kept in darkness for 10 min, after which the luciferase signals were monitored using the Tanon 5200 Chemiluminescent Imaging System (Tanon, Shanghai, China).

## Supplementary Material

qzaf050_Supplementary_Data

## Data Availability

The raw MS data were deposited into the ProteomeXchange Consortium with the iProX partner repository [73] (ProteomeXchange: PXD051570), which are publicly accessible at http://proteomecentral.proteomexchange.org.

## CRediT author statement

**Jinjuan Tan:** Conceptualization, Investigation, Data curation, Software, Writing – original draft. **Zhongjing Zhou:** Conceptualization, Validation. **Hanqian Feng:** Validation, Investigation. **Jiateng Zhang:** Validation, Investigation. **Ruikai Zhang:** Validation. **Zhongkai Chen:** Validation. **Yujie Niu:** Investigation. **Fangyu Liu:** Investigation. **Zhiping Deng:** Conceptualization, Methodology, Software, Formal analysis, Data curation, Writing – review & editing, Supervision, Project administration. All authors have read and approved the final manuscript.
