# Supplementary material for: Proteome and Phosphoproteome of Tomato Fruit Identify REDUCED CHLOROPLAST COVERAGE 1a as A Ripening Regulator
Source: Genomics Proteomics Bioinformatics. 2025 Jun 9;23(6):qzaf050. doi: 10.1093/gpbjnl/qzaf050 (PMC13220761; doi:10.1093/gpbjnl/qzaf050)
Supplement: qzaf050_Supplementary_Data [file qzaf050_supplementary_data.zip › supplementary material captions.docx]

**Supplementary materials**

**Figure S1 Distribution of differentially expressed proteins or phosphopeptides during ripening**

**A.** Violin plot showing differently expressed proteins or phosphopeptides during ripening. **B.** Proteins of various categories with significant alterations during ripening. Numbers in parentheses indicate the ratio of differentially expressed proteins to total identified proteins in each category.

**Figure S2 GO enrichment analysis of differentially expressed proteins at the protein abundance level**

GO, Gene Ontology; UDP, uridine diphosphate; snoRNA, small nucleolar RNA.

**Figure S3 KEGG enrichment analyses of differentially expressed proteins**

**Figure S4 GO enrichment analysis of differentially expressed proteins at the protein phosphorylation level**

**Figure S5 Motif analysis of the tomato phosphoproteome**

**A.** The proportion of proline-directed, acidic, basic, and other motif categories for specific phosphorylated residues. **B.** Motif category proportions for all identified phosphopeptides and upregulated phosphopeptides during ripening at the phosphopeptide and site-specific phosphorylation levels. Phospep, phosphopeptide; pS, phosphorylated serine; pT, phosphorylated threonine; pY, phosphorylated tyrosine.

**Figure S6 Modules assigned from the phosphoproteome based on WGCNA analysis**

Boxplots show the log abundance (ratio) of the eigenprotein of each module. Protein numbers clustered in each module are displayed at the top.

**Figure S7 KEGG pathway enrichment of differentially expressed proteins in WGCNA modules of the phosphoproteome**

**Figure S8 Module**–**trait relationship analysis for WGCNA modules in relation to pigment contents**

The Pearson correlation coefficients and *P* values (in parentheses) are displayed in each cell.

**Figure S9 CRISPR/Cas9-mediated gene editing of *REC1a***

**A.** Mutant position on the *REC1a* genomic sequence. **B.** Mutant bases. **C.** Sequence verification of the mutants. **D.** Off-target analysis. Seven potential off-target sites predicted by CRISPR-P 2.0 were verified using Sanger sequencing, and no off-target events were detected. CRISPR/Cas9, clustered regularly interspaced short palindromic repeats (CRISPR)-CRISPR-associated protein 9 (Cas9).

**Figure S10 Seeding, plant, and red ripe fruit phenotypes of *rec1a* mutants**

**A.** Seedling morphology. **B.** Seed germination ratio. **C.** Plant morphology. **D.** Flowering time. **E.** Fruit development period (from anthesis to BR stage). **F.** Lycopene and β-carotene contents in tomato fruits at BR+20. **G.** Fruit size. **H.** Total soluble solids content in tomato fruits at BR+20. Data are means ± SD, with asterisks indicating significant differences relative to the wild type (Student’s *t*-test, **, *P* < 0.01). BR+20, breaker + 20 days.

**Figure S11 Leaf phenotype of *rec1a* mutant**

**A.** Chlorophyll content in leaves of three-week-old plants. Data are presented as means ± SD of four biological replicates (Student’s *t*-test, **, *P* < 0.01). **B.** Individual mesophyll cells. **C–F.** Boxplots showing chloroplast coverage (**C**), chloroplast plan area (**D**), cell plan area (**E**), and chloroplast number per cell plan area (**F**), in *rec1a* mutants and wild type. Twenty-five cells were measured for each genotype, and *P* values (Student’s *t*-test) are displayed on top.

**Figure S12 Experimental workflow for TMT labeling-based proteomics analysis of *rec1a* mutant and wild-type fruits**

**Figure S13 CV distribution and PCA of *rec1a* and wild-type proteomes**

**A.** CV distribution among biological replicates of the proteome and the internal reference samples. **B.** PCA of the proteome of mutant and wild type. **C**. PCA analysis of the internal reference samples. CV, coefficient of variation.

**Figure S14 GO and KEGG enrichment analyses of differentially expressed proteins between *rec1a* mutant and wild type**

**Figure S15 Expression pattern of representative ripening-associated genes at protein abundance and transcript levels**

ClpB3, caseinolytic protease B3. Error bars indicate means ± SD of three (protein) or four (mRNA) biological replicates.

**Figure S16 Transcriptional expression of ripening-associated TFs and E3 ligases**

TFs and E3 ligases not detected in the TMT-based quantitative proteome analysis of *rec1a* mutant and wild-type fruits were analyzed. OFP20, OVATE Family Protein 20; BL4, bel1-like homeodomain 4; ARR1, response regulator 1; NAC4, NAM/ATAF1/2/CUC2 4; MYB72, myb domain protein 72; ZHD17, zinc-ﬁnger homeodomain protein 17; TKN, class I knotted1-like homeobox protein; GLK, Golden2-like; ARF, auxin response factor; FAS, fascinated; APRR2-like, ARABIDOPSIS PSEUDO RESPONSE REGULATOR2-like; BBX20, B-box zinc-finger transcription factor 20; SP1, suppressor of ppi1 locus1; SPL2, SP1-like 2. Error bars indicate means ± SD of four biological replicates.

**Figure S17 Eigengene**–**trait correlation analysis of *rec1a* mutant and wild-type proteomes**

The Pearson correlation coefficients and *P* values (in parentheses) are displayed in each cell.

**Figure S18 Proteins identified in this study and inconsistent expression patterns between proteins and mRNAs**

**A.** Venn diagram comparing proteins identified in this study with a reported transcriptome dataset [4]. **B.** Venn diagram comparing phosphopeptides (15-amino acid sequences centered around p-sites) with a previously reported tomato leaf phosphoproteome [57]. **C and D.** Heatmaps showing representative negative correlations (**C**) or no correlations (**D**) between protein and mRNA abundances. LR, light red; RR, red ripe.

**Figure S19 Phosphorylation of SR proteins during ripening**

**A.** Distribution of phosphorylation ratios at S/T/Y residues, with SR and SR-like proteins highlighted. **B.** Expression profiles of SR and SR-like proteins phosphorylation during ripening at site-specific levels. SR, serine/arginine-rich proteins [60].

**Figure S20** **Protein and mRNA levels of *REC1a* paralogs in ripening fruits of *rec1a* mutant and wild type**

Data are presented as means ± SD of three biological replicates (Student’s *t*-test, **, *P* < 0.01).

**Table S1 Proteome and phosphoproteome data from tomato (Ailsa Craig) fruits at different ripening stages**

**Table S2 Differentially expressed proteins and phosphoproteins during ripening, and REC1a phosphopeptide abundance determined by PRM**

**Table S3 Motif analysis of the phosphoproteome**

**Table S4 WGCNA of global proteins and phosphopeptides: Module assignment and ME-based connectivity**

**Table S5 Hub proteins and phosphopeptides within WGCNA modules identified from global proteome and phosphoproteome analyses of tomato fruit ripening**

**Table S6 Quantified fruit proteome of *rec1a* mutant and wild-type Micro-Tom**

**Table S7 Differentially expressed proteins between *rec1a* mutant and wild-type fruits**

**Table S8 Hub proteins in co-expression modules identified from *rec1a* mutant and wild-type proteome analyses**

**Table S9 Identification of REC1a-interacting proteins via co-IP and LC-MS/MS analysis**

**Table S10 Primers, sgRNA sequences, and isolation lists for PRM validation used in this study**
