# Supplementary figures and images for: Proteome and Phosphoproteome of Tomato Fruit Identify REDUCED CHLOROPLAST COVERAGE 1a as A Ripening Regulator
Source: Genomics Proteomics Bioinformatics. 2025 Jun 9;23(6):qzaf050. doi: 10.1093/gpbjnl/qzaf050 (PMC13220761; doi:10.1093/gpbjnl/qzaf050)

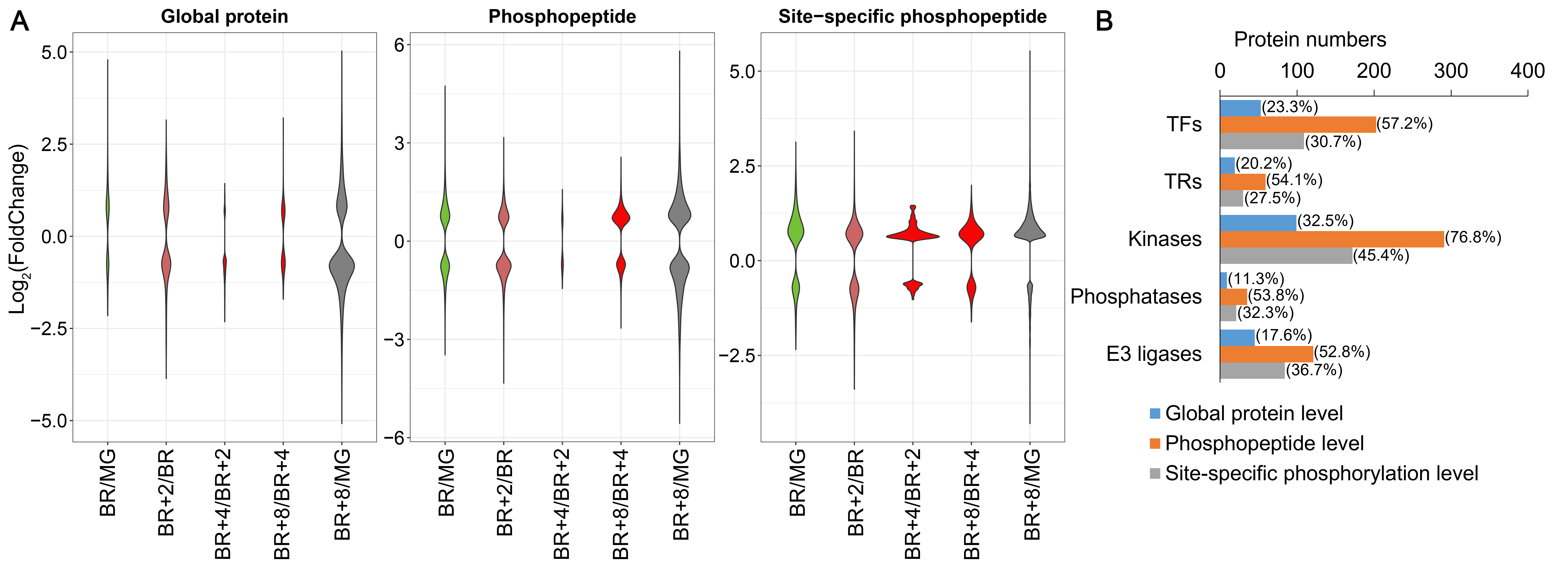

Supplement: qzaf050_Supplementary_Data [file qzaf050_supplementary_data.zip › Figure S1.tif]

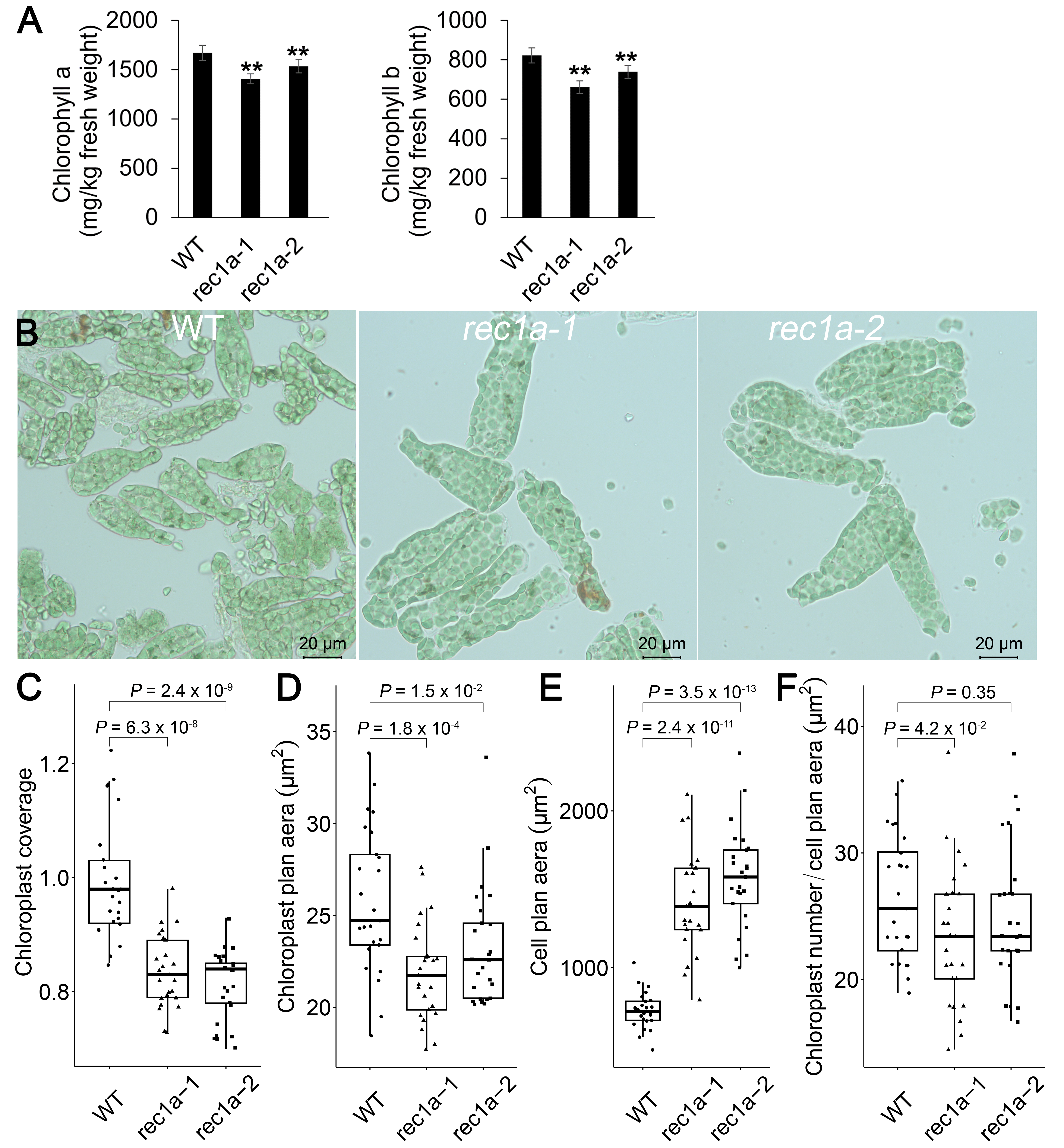

Supplement: qzaf050_Supplementary_Data [file qzaf050_supplementary_data.zip › Figure S11.tif]

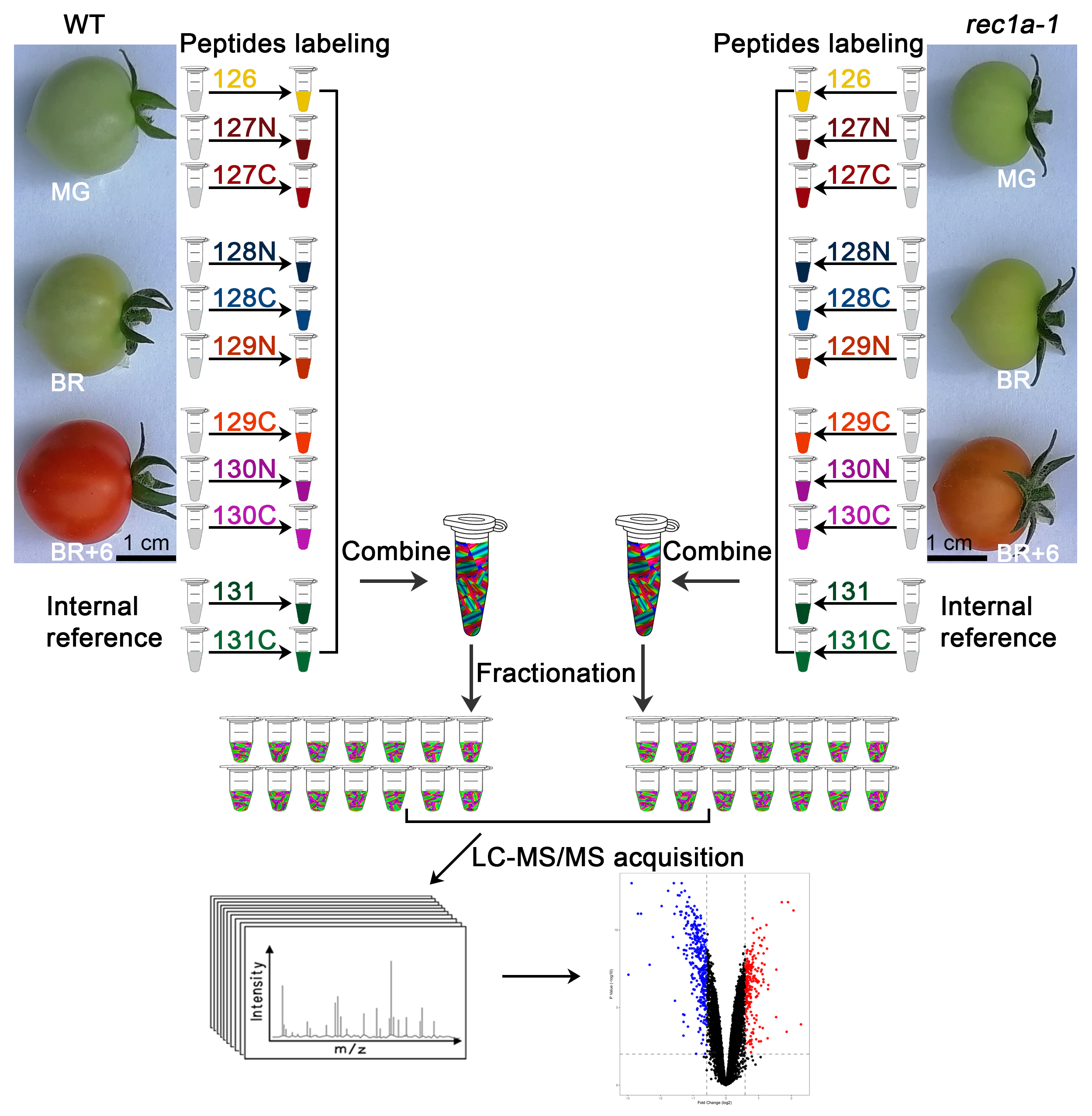

Supplement: qzaf050_Supplementary_Data [file qzaf050_supplementary_data.zip › Figure S12.tif]

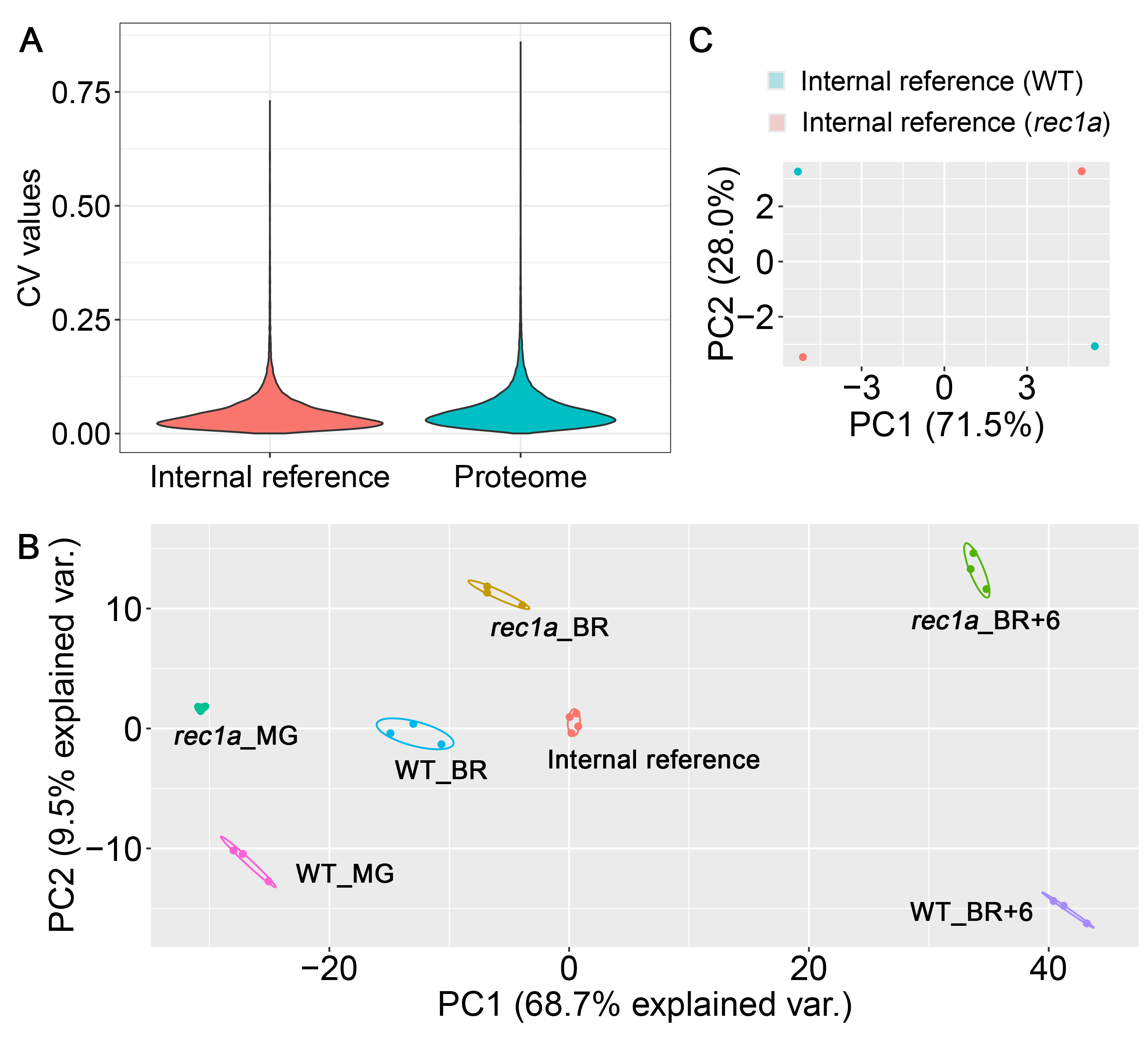

Supplement: qzaf050_Supplementary_Data [file qzaf050_supplementary_data.zip › Figure S13.tif]

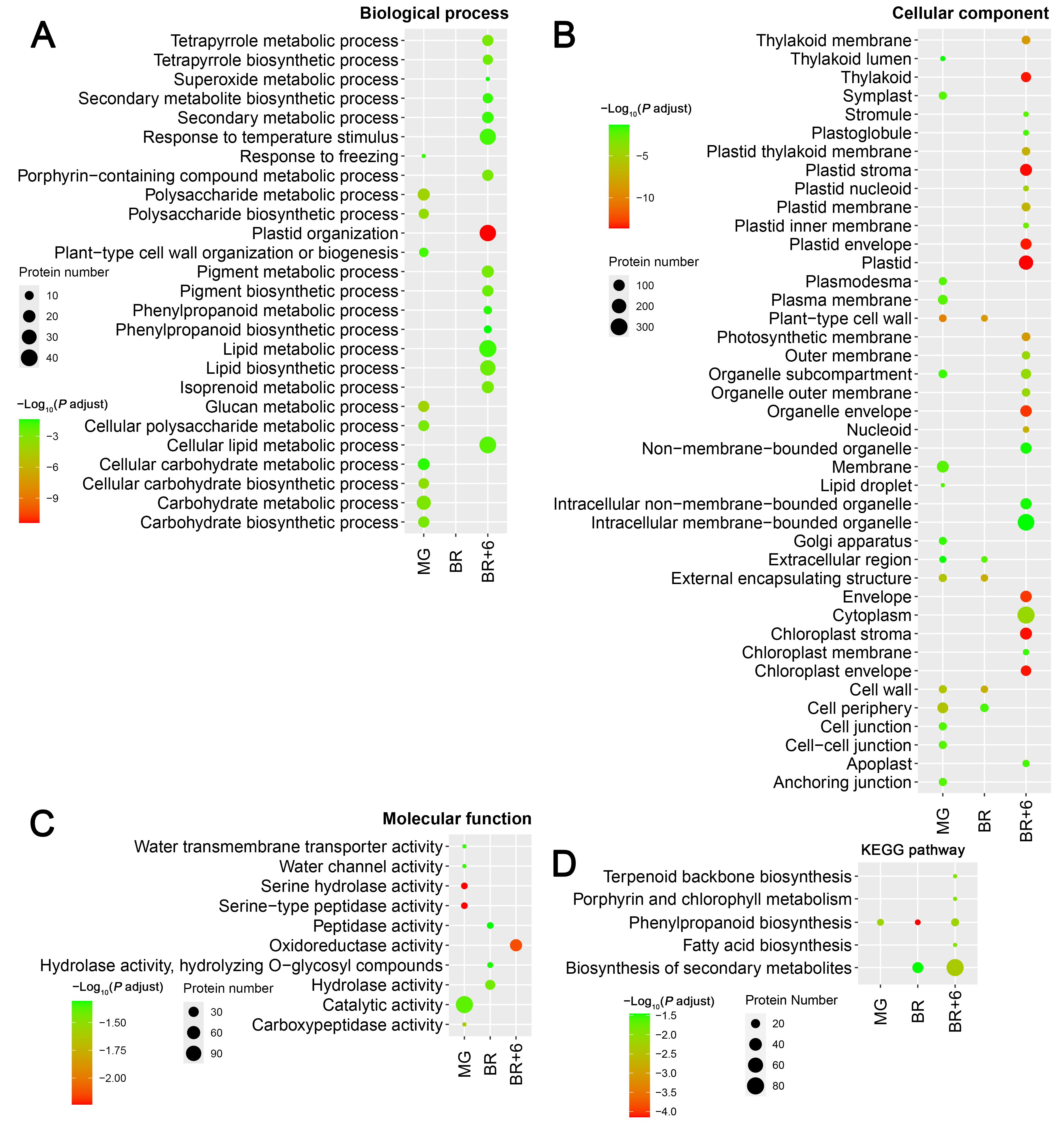

Supplement: qzaf050_Supplementary_Data [file qzaf050_supplementary_data.zip › Figure S14.tif]

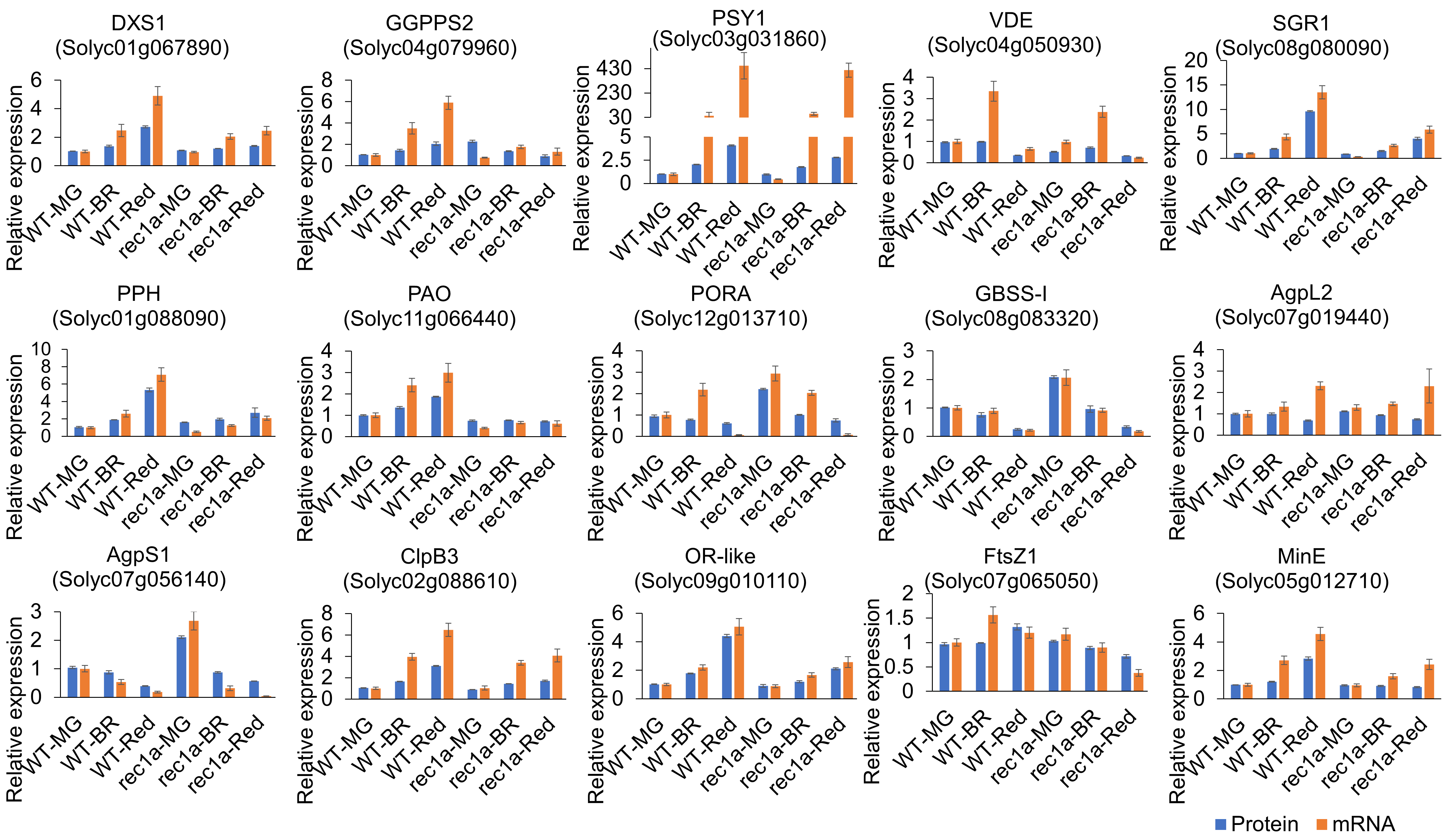

Supplement: qzaf050_Supplementary_Data [file qzaf050_supplementary_data.zip › Figure S15.tif]

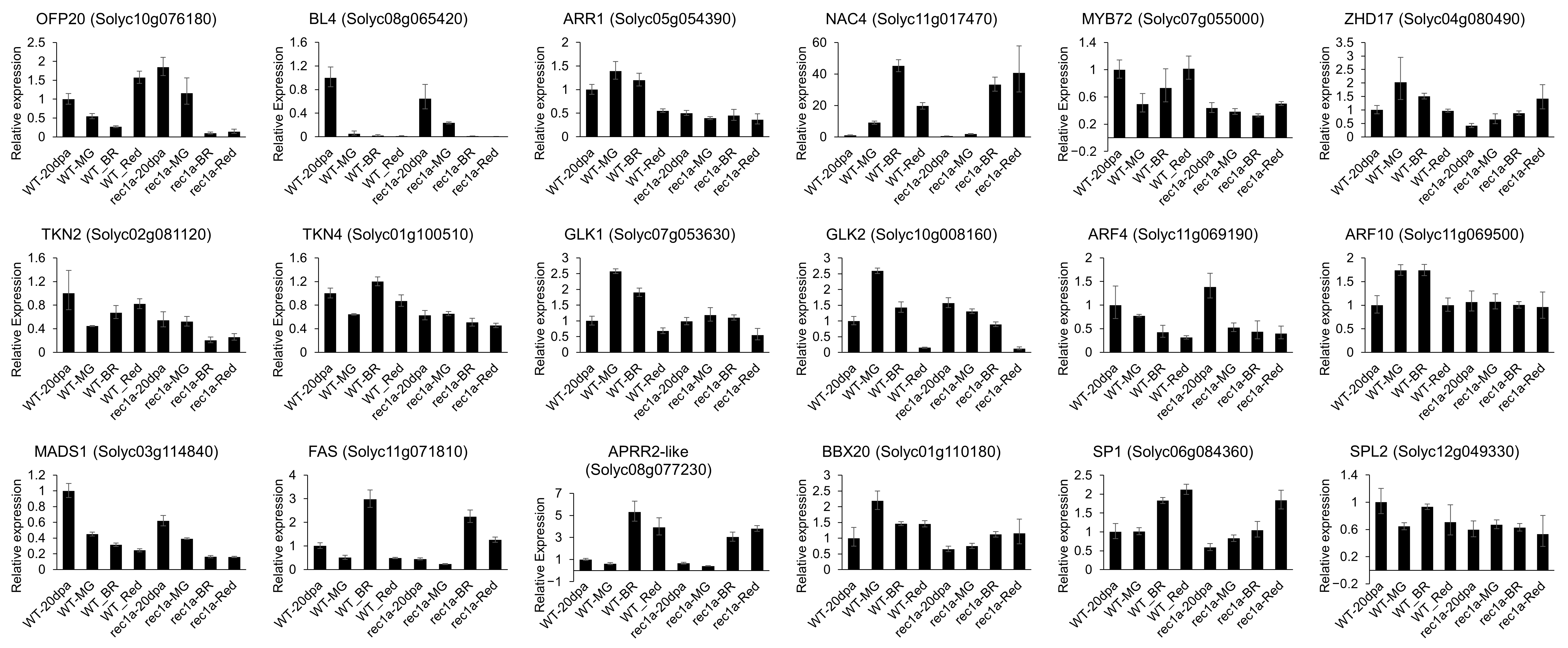

Supplement: qzaf050_Supplementary_Data [file qzaf050_supplementary_data.zip › Figure S16.tif]

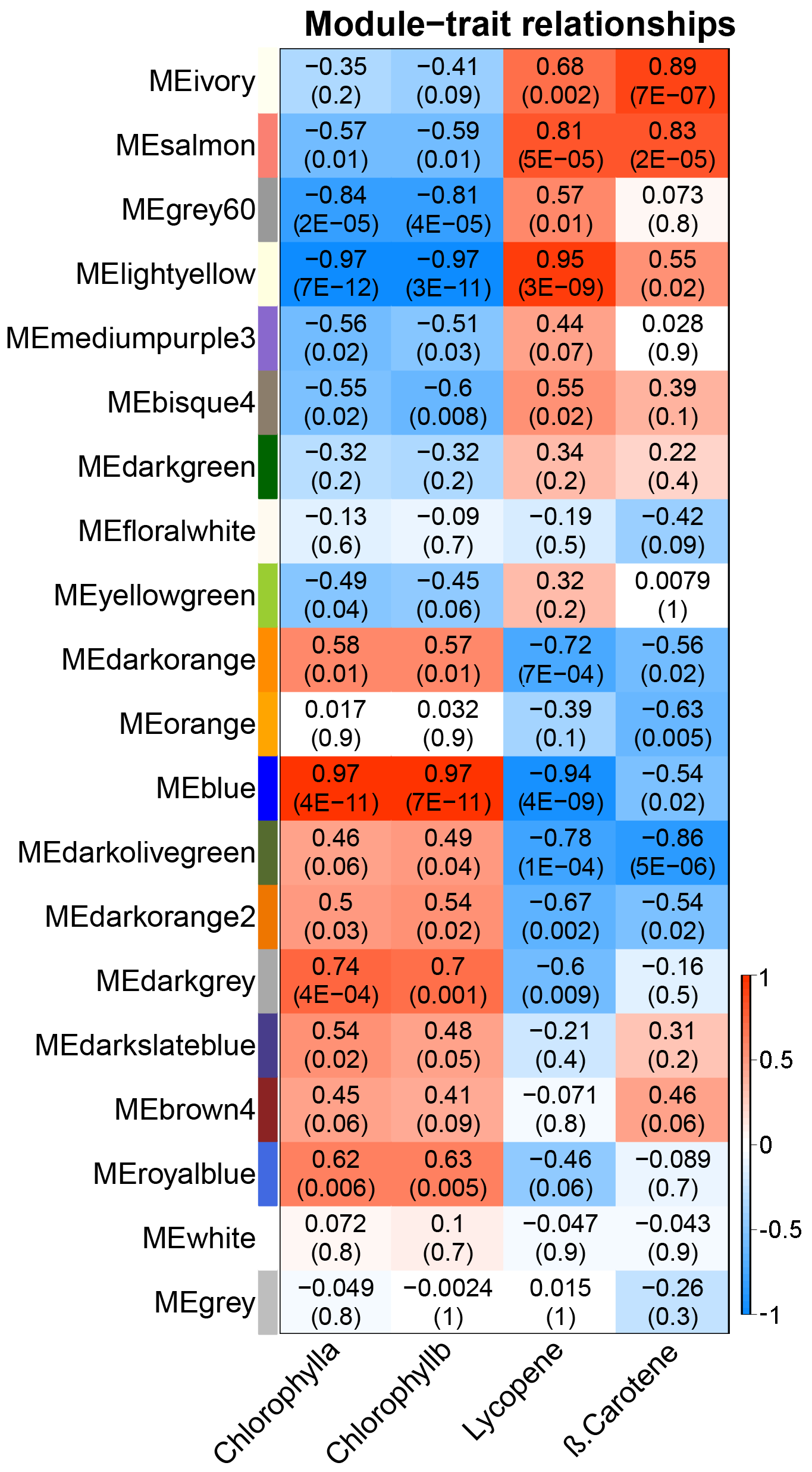

Supplement: qzaf050_Supplementary_Data [file qzaf050_supplementary_data.zip › Figure S17.tif]

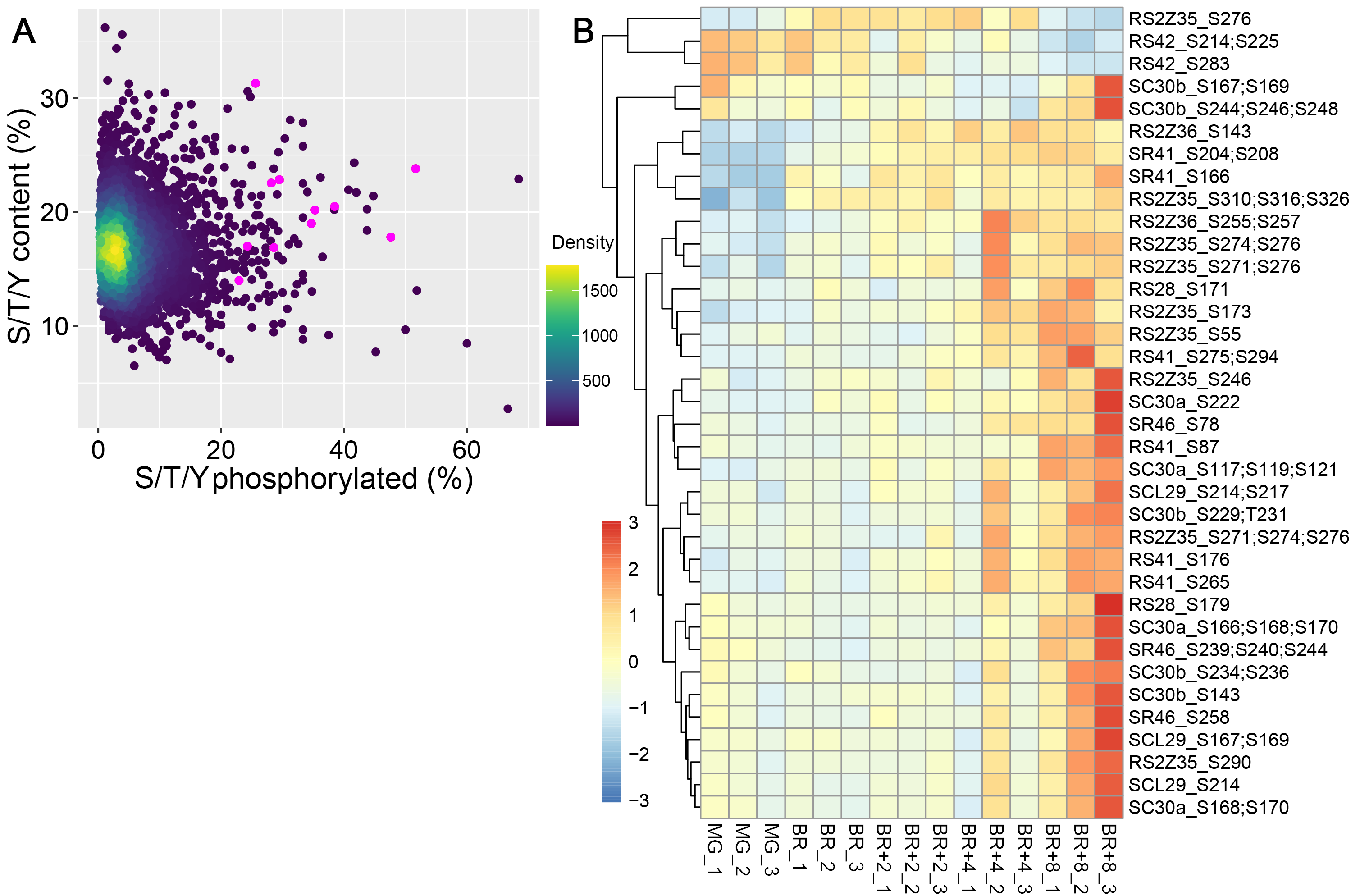

Supplement: qzaf050_Supplementary_Data [file qzaf050_supplementary_data.zip › Figure S19.tif]

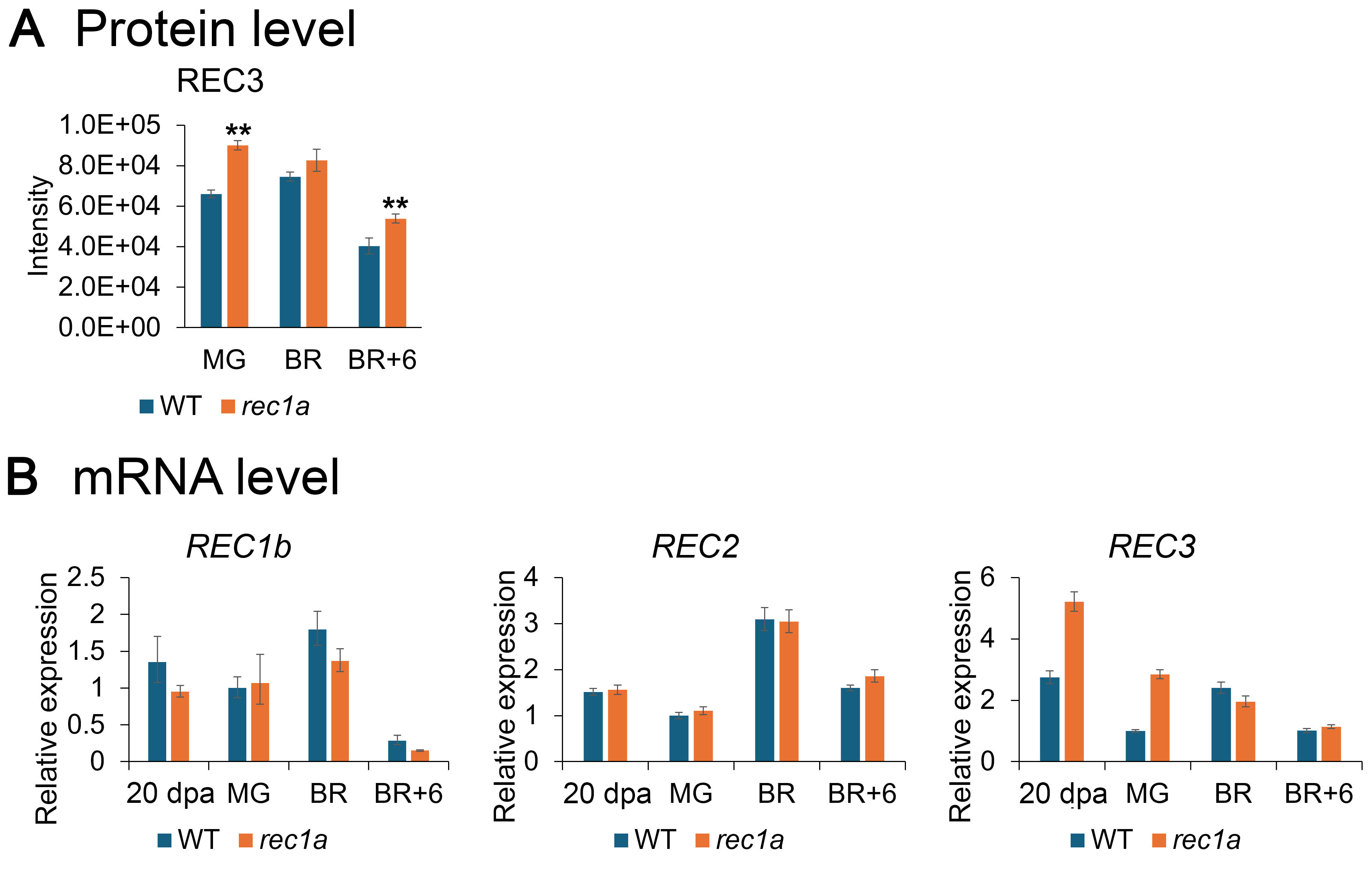

Supplement: qzaf050_Supplementary_Data [file qzaf050_supplementary_data.zip › Figure S20.tif]

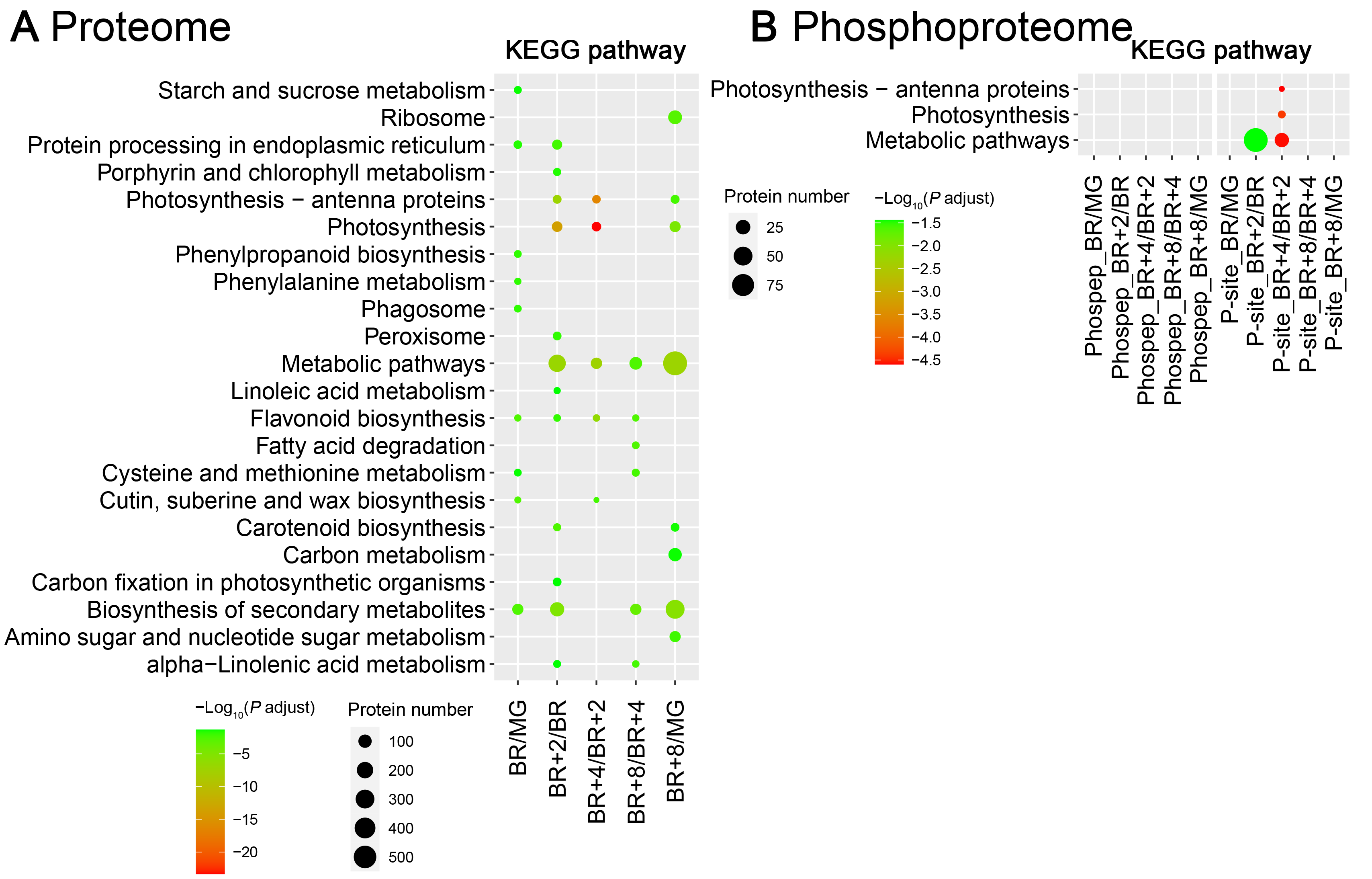

Supplement: qzaf050_Supplementary_Data [file qzaf050_supplementary_data.zip › Figure S3.tif]

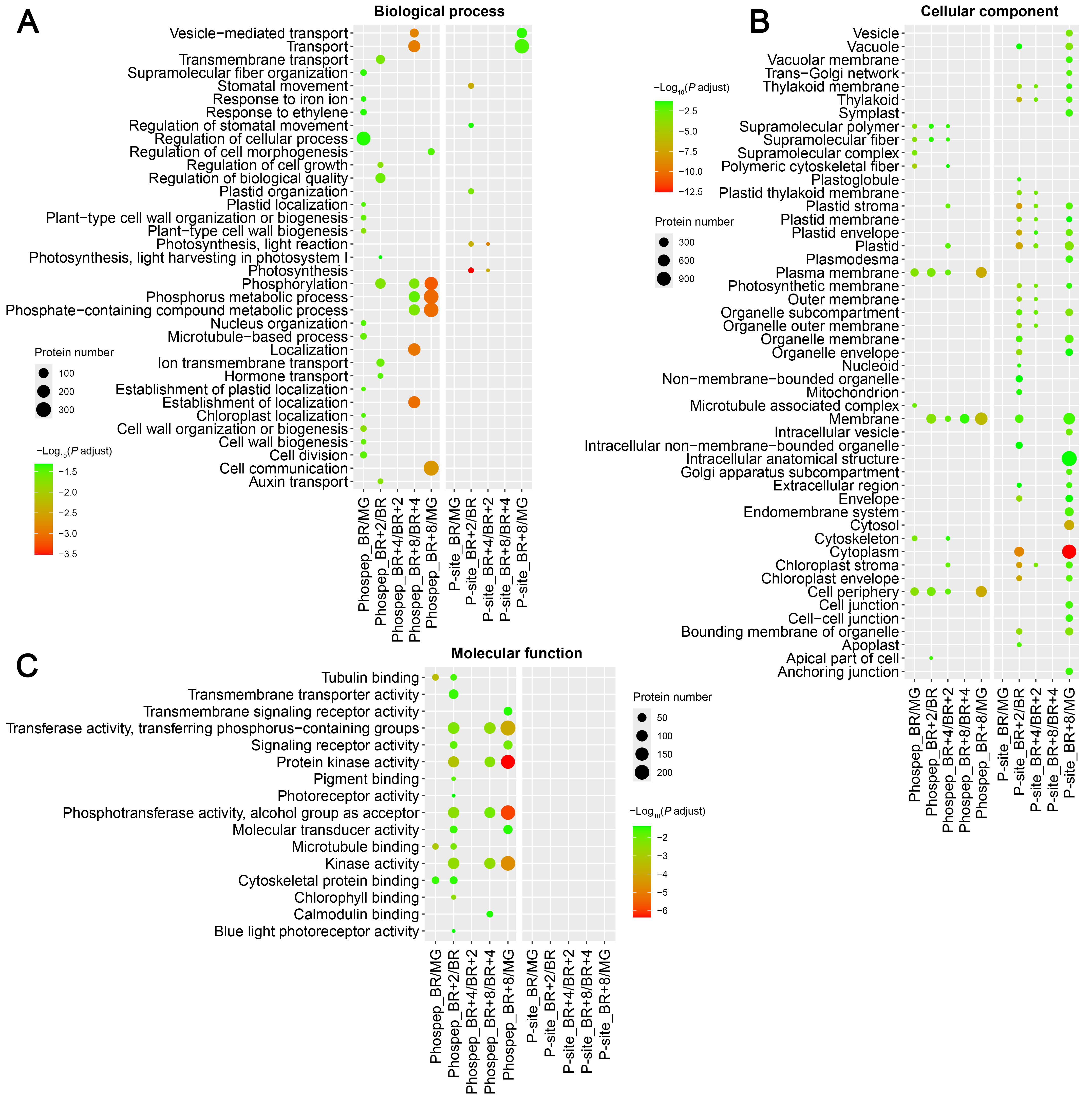

Supplement: qzaf050_Supplementary_Data [file qzaf050_supplementary_data.zip › Figure S4.tif]

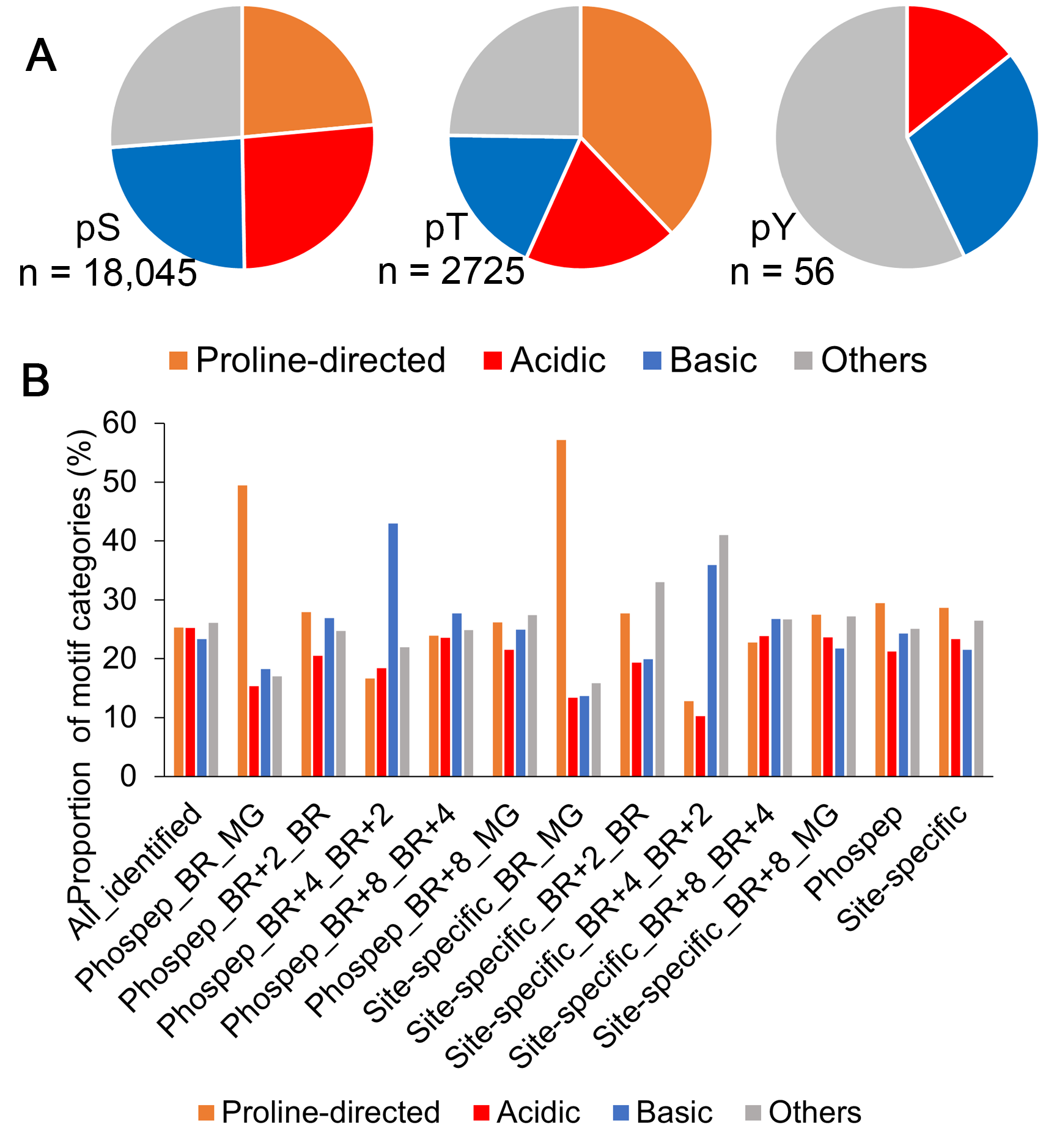

Supplement: qzaf050_Supplementary_Data [file qzaf050_supplementary_data.zip › Figure S5.tif]

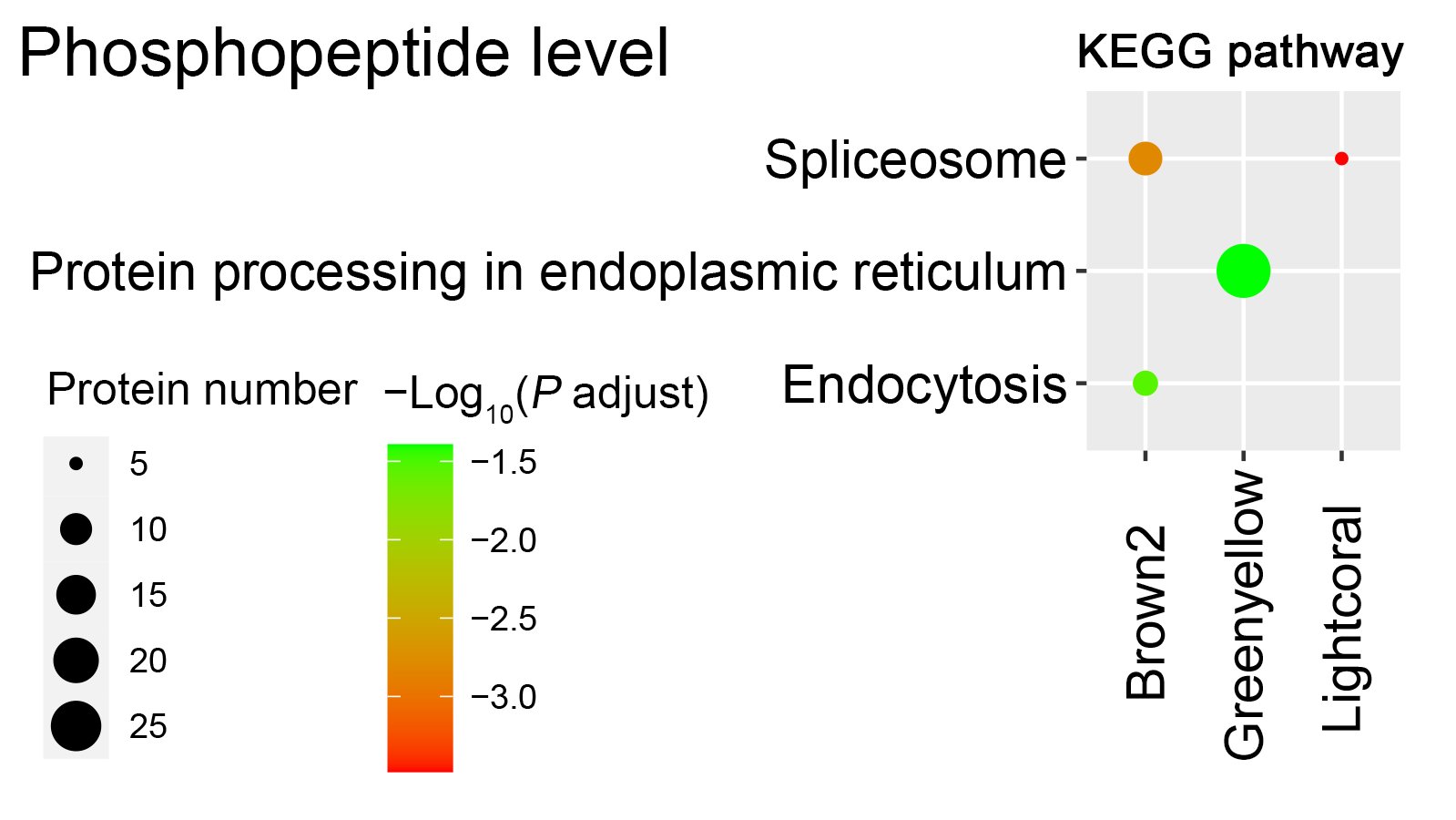

Supplement: qzaf050_Supplementary_Data [file qzaf050_supplementary_data.zip › Figure S7.tif]

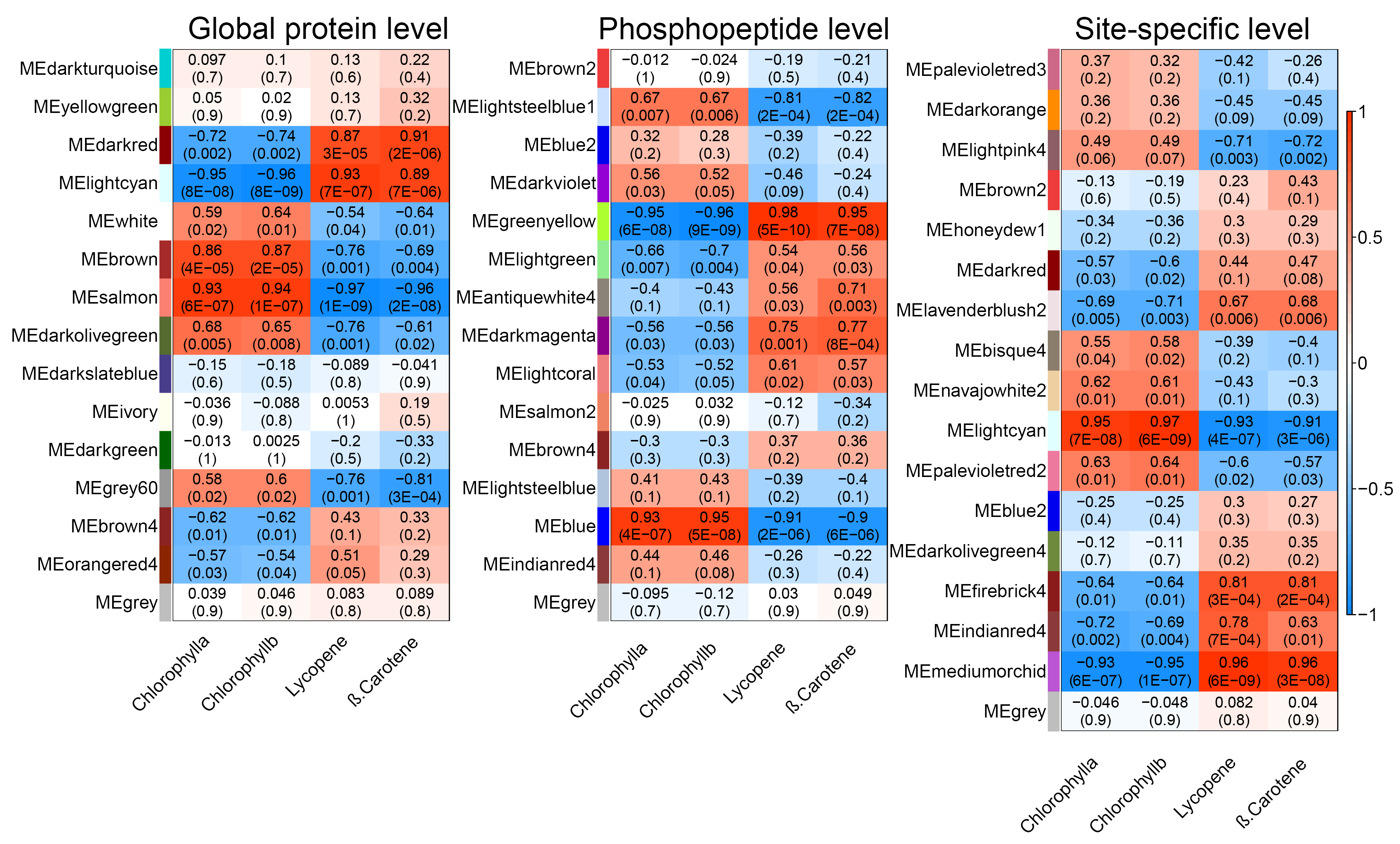

Supplement: qzaf050_Supplementary_Data [file qzaf050_supplementary_data.zip › Figure S8.tif]

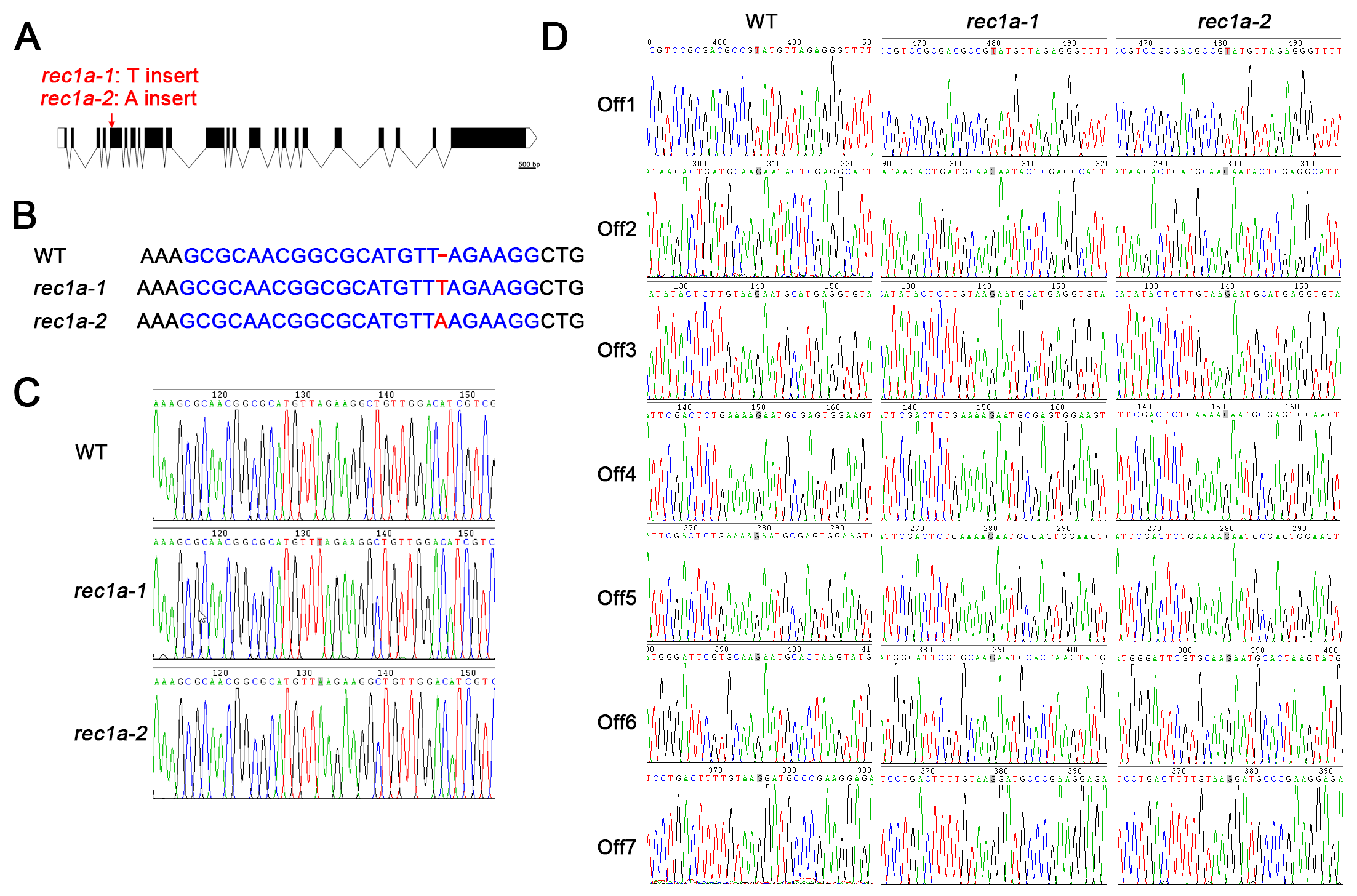

Supplement: qzaf050_Supplementary_Data [file qzaf050_supplementary_data.zip › Figure S9.tif]
